# Imaging-based diagnosis of sarcopenia for transplant-free survival in primary sclerosing cholangitis

**DOI:** 10.1186/s12876-024-03232-9

**Published:** 2024-04-25

**Authors:** Pedram Keshoofi, Philipp Schindler, Florian Rennebaum, Friederike Cordes, Haluk Morgul, Moritz Wildgruber, Hauke S. Heinzow, Andreas Pascher, Hartmut H. Schmidt, Anna Hüsing-Kabar, Michael Praktiknjo, Jonel Trebicka, Leon Louis Seifert

**Affiliations:** 1 Medical Clinic B, Department of Gastroenterology, Hepatology, Endocrinology, Infectiology, University Hospital Muenster, Albert-Schweitzer-Campus 1, Bldg. A14, 48149 Muenster, Germany; 2 Clinic for Radiology, University Hospital Muenster, 48149 Muenster, Germany; 3 Medical Clinic II, Euregio Hospital Nordhorn, 48529 Nordhorn, Germany; 4 Department for General, Visceral and Transplant Surgery, University Hospital Muenster, 48149 Muenster, Germany; 5 Department of Radiology, University Hospital LMU Munich, 81377 Munich, Germany; 6 Department of Internal Medicine I, Krankenhaus der Barmherzigen Brüder, 54292 Trier, Germany; 7 Department of Gastroenterology, Hepatology and Transplantation Medicine, University Hospital Essen, University of Duisburg-Essen, Hufelandstr. 55, 45147 Essen, Germany; 8 The Rockefeller University Center for Clinical and Translational Science, 10065 New York, NY United States of America; 9 Laboratory of Virology and Infectious Disease, The Rockefeller University, 10065 New York, NY United States of America; 10 Present Address: The Rockefeller University Hospital, 1230 York Avenue, 10065 New York, NY USA

**Keywords:** Skeletal muscle index, Chronic liver disease, Cirrhosis, meld, Standard exception criteria, Body mass index

## Abstract

**Background:**

Imaging-based assessment of sarcopenia is a well-validated prognostic tool for patients with chronic liver disease. However, little is known about its value in patients with primary sclerosing cholangitis (PSC). This cross-sectional study aimed to investigate the predictive value of the cross-sectional imaging-based skeletal muscle index (SMI) for transplant-free survival (TFS) in patients with PSC.

**Methods:**

A total of 95 patients with PSC who underwent abdominal cross-sectional imaging between 2008 and 2022 were included in this retrospective study. SMI was measured at the third lumbar vertebra level (L3-SMI). The cut-off values to define sarcopenia were < 50 cm²/m² in male patients and < 39 cm²/m² in female patients. The primary outcome of this study was TFS, which was defined as survival without liver transplantation or death from any cause.

**Results:**

Our study indicates that L3-SMI sarcopenia impairs TFS in patients with PSC (5-year TFS: 33.9% vs. 83.3%, *p* = 0.001, log-rank test). L3-SMI sarcopenia was independently associated with reduced TFS via multivariate Cox regression analysis (HR = 2.749; *p* = 0.028). Body mass index reduction > 10% at 12 months, which is used as MELD standard exception (SE) criterion in Eurotransplant (in Germany only until September 2023), was not significantly associated with TFS in the multivariate Cox regression analysis (HR = 1.417; *p* = 0.330). Substitution of BMI reduction with L3-SMI in the German SE criteria improved the predictive accuracy of TFS compared to the established SE criteria (multivariable Cox regression analysis: HR = 4.007, *p* < 0.001 vs. HR = 1.691, *p* = 0.141).

**Conclusion:**

Imaging-based diagnosis of sarcopenia via L3-SMI is associated with a low TFS in patients with PSC and may provide additional benefits as a prognostic factor in patient selection for liver transplantation.

**Supplementary Information:**

The online version contains supplementary material available at 10.1186/s12876-024-03232-9.

## Background

Determining the prognosis of patients with primary sclerosing cholangitis (PSC) is challenging. The natural history of patients with PSC is variable but frequently culminates in end-stage liver disease. The clinical course of PSC is complicated by an increased lifetime risk of up to 15% for cholangiocarcinoma (CCA) and, in case of concomitant inflammatory bowel disease (IBD), colorectal carcinoma (CRC) [1, 2]. Liver transplantation represents the only curative treatment option available.

The model of end-stage liver disease (MELD) score system is used as a tool to stratify disease severity in LTX patients on the waiting list, thus determining transplant priorities. However, MELD is not suitable for adequately determining the disease status and mortality in PSC [3, 4]. Therefore, standard exception (SE) criteria were implemented to depict disease severity in patients with PSC more appropriately and consequently alleviate underprioritizing of certain disease states [5]. According to the German Eurotransplant (ET) criteria (valid until 12th September 2023), SE status for PSC patients was granted if two out of the following three conditions applied: (1) recurrent cholangitis with ≥ 2 septic episodes over a span of 6 months, (2) detection of dominant bile duct stenosis, or (3) BMI decrease of > 10% within 12 months (General Eurotransplant SE criteria include detection of splenomegaly > 12 cm in place of a relevant bile duct stricture) [4, 6].

Sarcopenia refers to a state of progressive, generalized, and severe skeletal muscle wasting with impaired function, which can occur secondarily in patients with CLD [7]. A prevalence of as high as 50% has been reported in patients with advanced liver disease [8]. Sarcopenia has been linked to an overall impaired outcome with an increased risk of liver-related morbidity and mortality indicating its potential value as a prognostic tool pre- and post-LTX [8–10]. Among recently proposed imaging-based methods for the assessment of sarcopenia, the use of cross-sectional imaging at the third lumbar vertebrae layer (L3) to obtain skeletal muscle index (SMI) is well validated and is currently recommended by the EASL guidelines in patients with CLD [11–14].

Despite the use of SE criteria, recent studies have pointed to a lack of medical progress in the outcome of patients with PSC after LTX [3]. Coincidentally, as of January 2023, the German Medical Association released new guidelines including revised SE criteria for PSC patients that entered into force on 12th September and will be addressed further below [15]. Nonetheless, reconsidering established liver allocation policies and addressing the unmet need for well-validated and objective prognostic tools for patients with PSC are required [3, 16].

To date, there are no criteria based on imaging analysis of sarcopenia in patients with PSC. This study aimed to provide further insight into the relevance of imaging-based diagnosis of sarcopenia as an independent risk factor in patients with PSC and its implications for patient management.

## Methods

### Study Design

The primary outcome of this retrospective, cross-sectional study was to determine the impact of sarcopenia on transplant-free survival (TFS) at 1, 2 and 5 years following cross-sectional imaging. The mean time interval between the initial diagnosis of PSC and the date of imaging was 9 years (SD 7.5 years). TFS was defined as survival without liver transplantation or death from any cause.

### Data collection

**Fig. 1:**
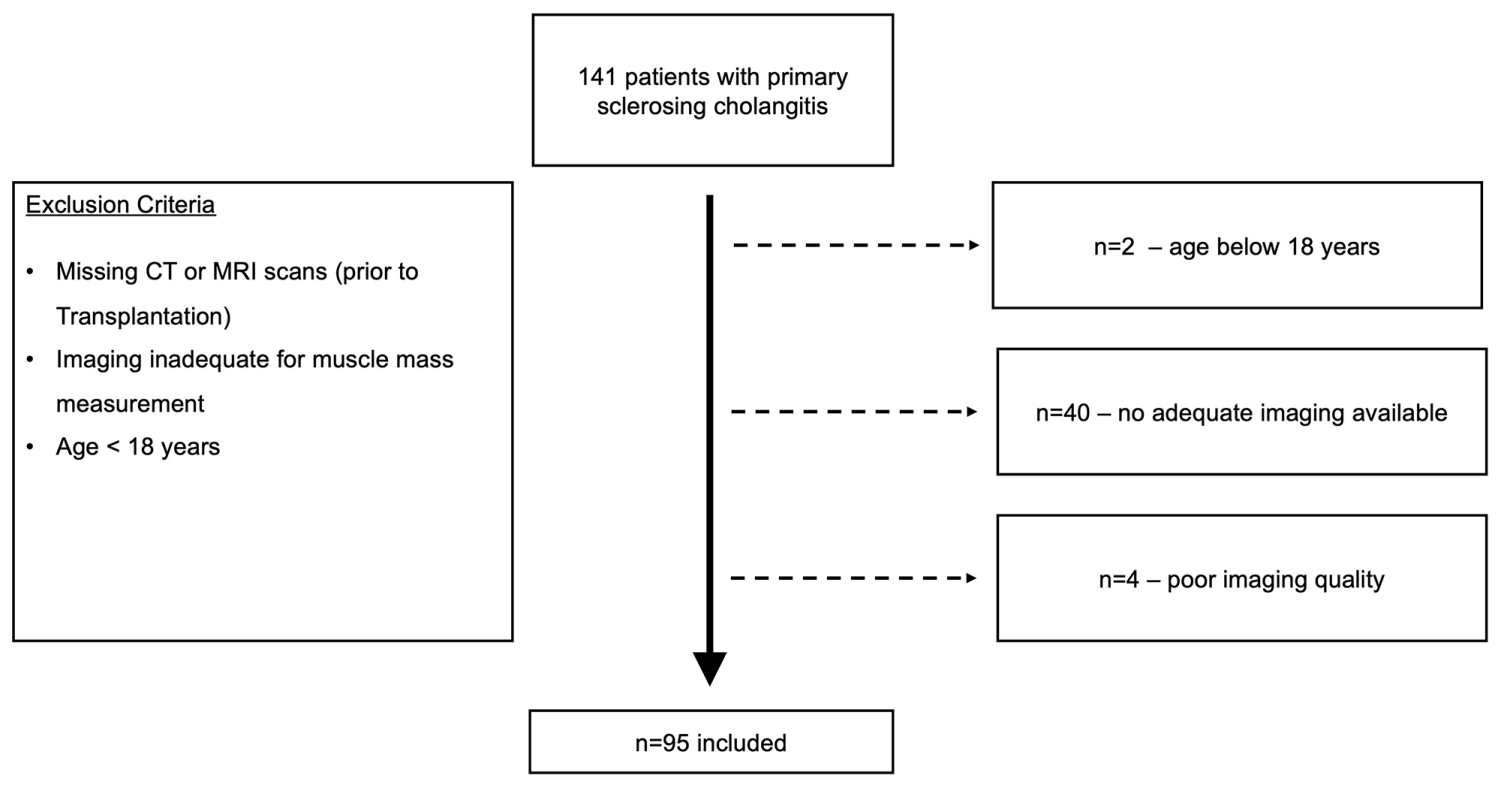
Flow chart of patient selection and exclusion criteria

This retrospective, single-center study included patients with PSC registered at a German tertiary university liver center. All study procedures conformed to the declaration of Helsinki and were approved by the local ethics committee (file number: 2018-378-f-S). The requirement for patient consent was waived, owing to the retrospective nature of the study. Patient data were collected via electronic review of patient records. Data from a total of 141 patients with PSC were available. PSC was diagnosed clinically, through either endoscopic retrograde cholangiopancreatography or magnetic resonance cholangiopancreatography (ERCP/MRCP), and/or histologically in the absence of identifiable reasons for secondary sclerosing cholangitis [17]. The patient selection criteria were installed for further analysis (Fig. 1).

The inclusion criteria were diagnosis of PSC and availability of abdominal CT or MRI scans. Patients were excluded if they were below the age of 18 years, had missing CT/MRI scans, or muscle mass analysis was infeasible due to inadequate imaging quality. Out of the initial 141 patients, 46 were excluded from the study.

All included patients were treated according to the German SE criteria for patients with PSC who underwent liver transplantation.

Given that the observational period of this retrospective study spans from 2008 to 2022, the former German SE criteria were applied. Consequently, primary statistical analysis focuses on the German PSC SE criteria used at that time.

Baseline patient characteristics are presented in Table 1. The Child-Pugh score, Mayo risk score, EASL 2022 Clinical Practice Guidelines approach to risk stratification, and MELD score were evaluated based on clinical data. Follow-up data were retrieved until death, LTX, or end of/loss to follow-up. Loss to follow-up was defined as failure to return to the healthcare facility for care or treatment refill for more 360 days from the previous visit.

**Table 1:** Baseline characteristics of patients grouped by sarcopenia

Parameters	All Patients	Sarcopenia	No-sarcopenia	*p*-value
(N) = available cases if values are missing	% (total number) or median/mean (range/SD)			
Number of patients	95	62.1% (59)	37.9% (36)	–
Sex				0.310
Male	67.4% (64)	71.2% (42)	61.1% (22)	
Female	32.6% (31)	28.8% (17)	38.9% (14)	
Age (median, range, in years)	45 (18–76)	46 (18–76)	41 (23–64)	0.369
BMI	25.7 (7.7)	24.8 (8.1)	27.1 (6.9)	0.167
PSC-IBD	70.5% (67)	66.1% (39)	77.8% (28)	0.226
Liver cirrhosis	51.6% (48)	61.0% (36)	33.3% (12)	0.017
LTX	35.8% (34)	47.5% (28)	16.7% (6)	0.002
Death	11.6% (11)	18.6% (11)	0% (0)	0.006
LTX/death (primary endpoint)	47.4% (45)	66.1% (39)	16.7% (6)	< 0.001
Malignancy	20.0% (19)	25.4% (15)	11.1% (4)	0.091
Dominant stenosis	67.4% (64)	69.5% (41)	61.9% (23)	0.572
Ascites	40.0% (38)	54.2% (32)	16.7% (6)	< 0.001
Hepatic Encephalopathy	13.7% (13)	16.9% (10)	8.3% (3)	0.236
Splenomegaly	48.4% (46)	57.6% (34)	33.3% (12)	0.022
BMI reduction > 10% in 12 months	16.8% (16)	22.0% (13)	8.3% (3)	0.083
Recurring cholangitis (≥ 2x/6 months)	16.8% (16)	22.0% (13)	8.3% (3)	0.083
SEG	27.4% (26)	37.3% (22)	11.1% (4)	0.006
MELD score (*n* = 94)	13.1 (8.2)	14.9 (8.7)	9.9 (6.2)	0.002
Child-Pugh Grade (*n* = 48)				0.001
A	25.0% (12)	13.9% (5)	58.3% (7)	
B	50.0% (24)	55.6% (20)	33.3% (4)	
C	25.0% (12)	30.6% (11)	08.3% (1)	
Mayo risk score (*n* = 67)	1.70 (1.87)	2.27 (1.81)	0.62 (1.52)	<0.001
EASL 2022 CPG risk stratification				< 0.001
Low risk	22.1% (21)	15.3% (9)	33.3% (12)	
Significant risk	77.9% (74)	84.7% (50)	66.7% (24)	
Platelets (10^3^cells/µl)	227 (149)	231 (169)	219 (110)	0.708
Albumin (g/dl) (*n* = 67)	3.64 (0.86)	3.36 (0.86)	4.19 (0.57)	< 0.001
Bilirubin (mg/dl)	5.0 (7.7)	6.5 (8.8)	2.7 (4.7)	0.019
INR (*n* = 90)	1.21 (0.41)	1.29 (0.45)	1.07 (0.32)	0.012
Creatinine (mg/dl)	0.9 (0.5)	0.9 (0.5)	0.9 (0.6)	0.855
Sodium (mmol/l) (*n* = 90)	138 (4)	137 (5)	140 (2)	< 0.001
Mode of cross-sectional imaging				0.070
CT	58.9% (56)	66.1% (39)	47.2% (17)	
MRI	41.1% (39)	33.9% (20)	52.8% (19)	
Time interval between diagnosis of PSC and date of last imaging (years)	8.89 (7.52)	8.95 (7.71)	8.81 (7.32)	0.929
Loss to follow-up	23.2% (22)	15.3% (9)	36.1% (13)	0.019
Time of follow-up (years)	1.35 (2.06)	1.06 (1.66)	1.82 (2.53)	0.078
SMI (cm²/m²)	42.08(9.35)	37.55 (7.63)	49.49 (6.31)	< 0.001

Abbreviations: BMI, body mass index; CT, computed tomography; EASL 2022 CPG, European Association for the Study of the Liver 2022 Clinical Practice Guidelines; INR, international normalized ratio; LTX, liver transplantation; MELD, Model of End-Stage Liver Disease; MRI, magnetic resonance imaging; PSC-IBD, patient with primary sclerosing cholangitis and associated inflammatory bowel disease: SEG, Standard Exception criteria of Germany; SMI, skeletal muscle index

### Imaging analysis

The latest available abdominal cross-sectional imaging data were analyzed for every patient to ensure high quality of imaging and standardized imaging selection. The mean interval between the initial diagnosis of PSC and the date of imaging was nine years (SD 7.5 years). Muscle mass measurements were obtained on axial CT/MRI scans at the level of the third lumbar vertebrae (L3) by using the analytic morphomics software *Coreslicer*. The L3 region contains the following muscles: the psoas, paraspinal muscles (erector spinae, quadratus lumborum), and abdominal wall muscles (transversus abdominus, external and internal obliques, and rectus abdominus) [14]. The skeletal muscle and adipose tissue areas were automatically differentiated and segmented by the software. The total skeletal muscle surface area was semi-automatically calculated using the software and double-checked by the operator (Supplementary Fig. 1). The skeletal muscle index (SMI) was calculated based on the resulting numerical values. SMI was defined as the sum of the cross-sectional area of all previously mentioned muscles on a single scan at L3, which was normalized by the height (m) of the patient [18, 19].

$$ \begin{array}{l}Skeletal\,muscle\,index\left( {SMI} \right)at\,L3 = \\\,\,\,\,\,\,\frac{{Total\,skeletal\,muscle\,area\,\left( {TSMA} \right)\,at\,L3\,c{m^2}}}{{height\,\left( m \right)\, \times \,height\,\left( m \right)}}\end{array}$$

All measurements were performed in a blinded manner. Established cut-off values for L3-SMI (< 50 cm²/m² in male patients; <39 cm²/m² in female patients) were used, as recommended by the EASL guidelines, and validated by multiple recent studies on cirrhotic patients [12, 13, 20–22].

### Statistical analysis

Statistical analyses were performed using *SPSS* (IBM Corp. Released 2021. IBM SPSS Statistics for Macintosh, Version 28.0.1.0 Armonk, NY: IBM Corp) and the *cmprsk* package in *R* version 4.3.1 (R Core Team (2023). R: A language and environment for statistical computing. R Foundation for Statistical Computing, Vienna, Austria: https://www.r-project.org).

All continuous variables are appropriately displayed as the mean and standard deviation (SD) or median and range. Categorical variables are presented as numbers and/or percentages. The Student’s t-test was used for parametric data and the Mann-Whitney-U test for non-parametric data when comparing differences among groups for continuous variables. Chi-square tests, including the Phi-coefficient and Cramer’s V, were used for group comparisons of categorical variables.

To identify independent risk factors associated with impaired 5-year TFS after the last cross-sectional imaging, univariate Cox regression analyses were performed on the baseline characteristics (Table 2). All variables related to the pathogenetic mechanism of PSC and significantly associated with TFS (*p* < 0.05) were consecutively included in the multivariate regression analysis (Table 2). To avoid overlap in variables and maintain an adequate ratio of events per variable (EPV), variables that consisted of multiple single factors, such as SE status or MELD score, were not included in the calculation but only their single components if they were significantly associated with TFS.

**Table 2:** Characteristics associated with 5-year transplant-free survival by Cox regression analysis in patients with primary sclerosing cholangitis

Parameter	Univariate model		Multivariate model	
	HR (95% CI)	*p*-value	HR (95% CI)	*p*-value
Sarcopenia via L3-SMI	5.561 (2.346–13.182)	< 0.001	2.749 (1.118–6.758)	0.028
Sex	0.607 (0.307–1.198)	0.150		
Age	1.009 (0.988–1.032)	0.400		
BMI	1.015 (0.982–1.048)	0.377		
PSC-IBD	0.838 (0.451–1.559)	0.577		
Liver cirrhosis	10.870 (4.714–25.070)	< 0.001		
Malignancy	1.457 (0.736–2.883)	0.280		
Dominant Stenosis	2.930 (1.359–6.317)	0.006	1.237 (0.516–2.961)	0.634
Ascites	6.528 (3.426–12.442)	< 0.001		
Hepatic Encephalopathy	2.175 (1.074–4.405)	0.031		
Splenomegaly	2.948 (1.581–5.498)	< 0.001	2.473 (1.203–5.087)	0.014
BMI reduction > 10% in 12 months	3.106 (1.636–5.894)	< 0.001	1.417 (0.703–2.856)	0.330
Recurring cholangitis (≥ 2x/6 months)	2.711 (1.424–5.161)	0.002	2.529 (1.190–5.376)	0.016
SEG	3.860 (2.122–7.021)	< 0.001		
MELD score	1.121 (1.088–1.156)	< 0.001		
Child-Pugh Grade	3.727 (2.302–6.036)	< 0.001		
Mayo risk score	1.589 (1.325–1.905)	< 0.001		
EASL 2022 CPG risk stratification	3.904 (1.396–10.919)	0.009		
Platelets	0.997 (0.994–0.999)	0.015		
Albumin	0.419 (0.307–0.571)	< 0.001		
Bilirubin	1.114 (1.078–1.151)	< 0.001	1.079 (1.023–1.137)	0.005
INR	4.497 (2.720–7.435)	< 0.001	1.560 (0.673–3.617)	0.300
Creatinine	1.567 (1.018–2.411)	0.041		
Sodium	0.894 (0.852–0.938)	< 0.001		
Mode of cross-sectional imaging	0.342 (0.173–0.675)	0.002		
SMI	0.973 (0.943–1.004)	0.083		

Abbreviations: BMI, body mass index; EASL 2022 CPG, European Association for the Study of the Liver 2022 Clinical Practice Guidelines; INR, international normalized ratio; LTX, liver transplantation; MELD, Model of End-Stage Liver Disease; PSC-IBD, patient with primary sclerosing cholangitis and associated inflammatory bowel disease: SEG, Standard Exception criteria of Germany; SMI, skeletal muscle index

With regard to clinical data on the signs of CLD, we selected splenomegaly as a representative indicator of portal hypertension for inclusion in the multivariable Cox analysis, as it represents a Eurotransplant SE criterion in patients with PSC. Ultimately multivariable Cox regression was performed using the following covariates: L3-SMI sarcopenia, dominant stenosis, splenomegaly, BMI reduction of > 10% in 12 months, recurrent cholangitis with ≥ 2 septic episodes within 6 months, bilirubin level, and INR (Table 2).

Estimation of survival related to the impact of sarcopenia was performed by comparison with the log-rank test and illustrated using Kaplan-Meier curves. A competing-risk analysis was performed to further assess the association between L3-SMI sarcopenia and mortality using LTX as a competing risk. Statistical significance for 2-sided tests was set at *p* < 0.05.

## Results

### Patient characteristics

95 patients with available abdominal cross-sectional imaging for sarcopenia analysis were included after meeting the selection criteria (Fig. 1). The baseline characteristics of the patients are summarized in Table 1 (and supplementary Table 1). The median age at the time of cross-sectional imaging was 45 (18–76) years and the patient cohort consisted of 67.4% (64) male patients and 32.6% (31) female patients. L3-SMI-based sarcopenia was diagnosed in 62.1% (59) of patients. Liver cirrhosis was more prevalent in patients with sarcopenia than in those without (61.0% vs. 33.3%; *p* = 0.017). The mean MELD score was 13 (standard deviation [SD] = 8.2). A higher mean MELD score was observed in patients with sarcopenia (15; SD = 8.7) than in those without sarcopenia (10; SD = 6.2). No significant difference in BMI was found between patients with and without sarcopenia (mean BMI of sarcopenic patients 24.8 vs. non-sarcopenic patients 27.1; *p* = 0.167).

### Risk factors of impaired transplant-free survival

Overall, 52.6% (*n* = 50) of patients achieved transplant-free survival during the 5-year observation period. Correspondingly, 35.8% (*n* = 34) of patients received LTX and 11.6% (*n* = 11) died, amounting to 47.4% (*n* = 45) patients who met the primary outcome of this study (LTX or death). Univariate Cox regression analyses were performed to identify risk factors for impaired TFS (Table 2 and Supplementary Tables 2 and 3). Subsequently, using multivariate Cox regression analysis, we confirmed that sarcopenia was independently associated with reduced transplant-free survival (hazard ratio [HR] = 2.749; *p* = 0.028). Splenomegaly (HR = 2.473; *p* = 0.014), recurring cholangitis (HR = 2.529; *p* = 0.016), and bilirubin level (HR = 1.079; *p* = 0.005) were identified as additional risk factors. Despite a significant correlation in the univariate analysis, BMI reduction > 10% at 12 months was not significantly associated with TFS in the multivariate model applied (HR = 1.417; *p* = 0.330; Table 2).

### Sarcopenia and 5-year transplant-free survival

**Fig. 2:**
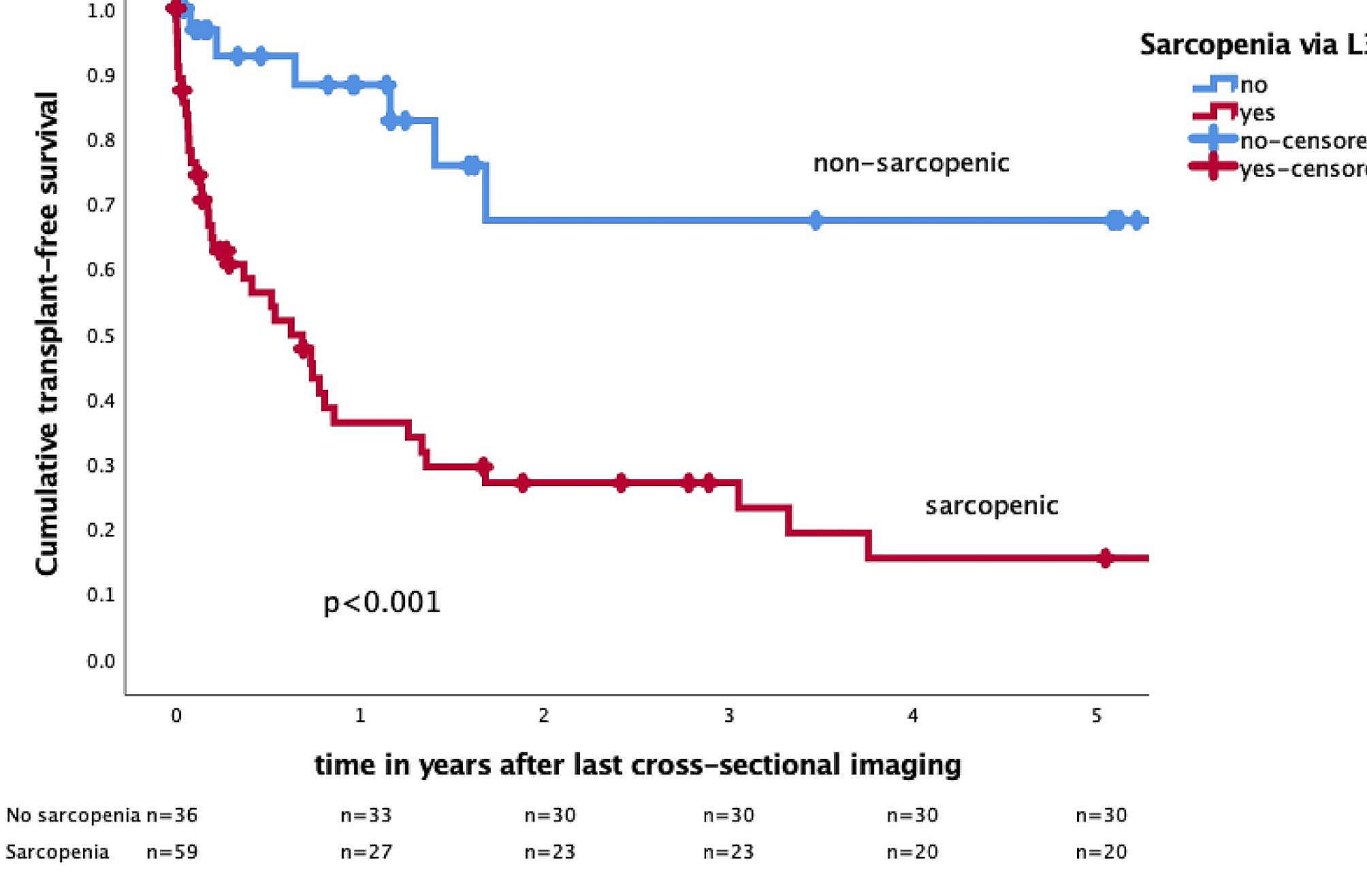
Transplant-free survival over 5 years after imaging in patients with and without sarcopenia indicated by Kaplan-Meier curves Transplant-free survival of *n* = 95 patients with PSC at 5 years after last cross-sectional imaging relative to sarcopenia determined by skeletal muscle index at vertebra L3 (L3-SMI). Transplant-free survival 5 years after last imaging was at 83.3% for patients without sarcopenia compared to 33.9% of patients with detected sarcopenia (log-rank test *p* < 0.001). Censored patients shown as +

**Fig. 3:**
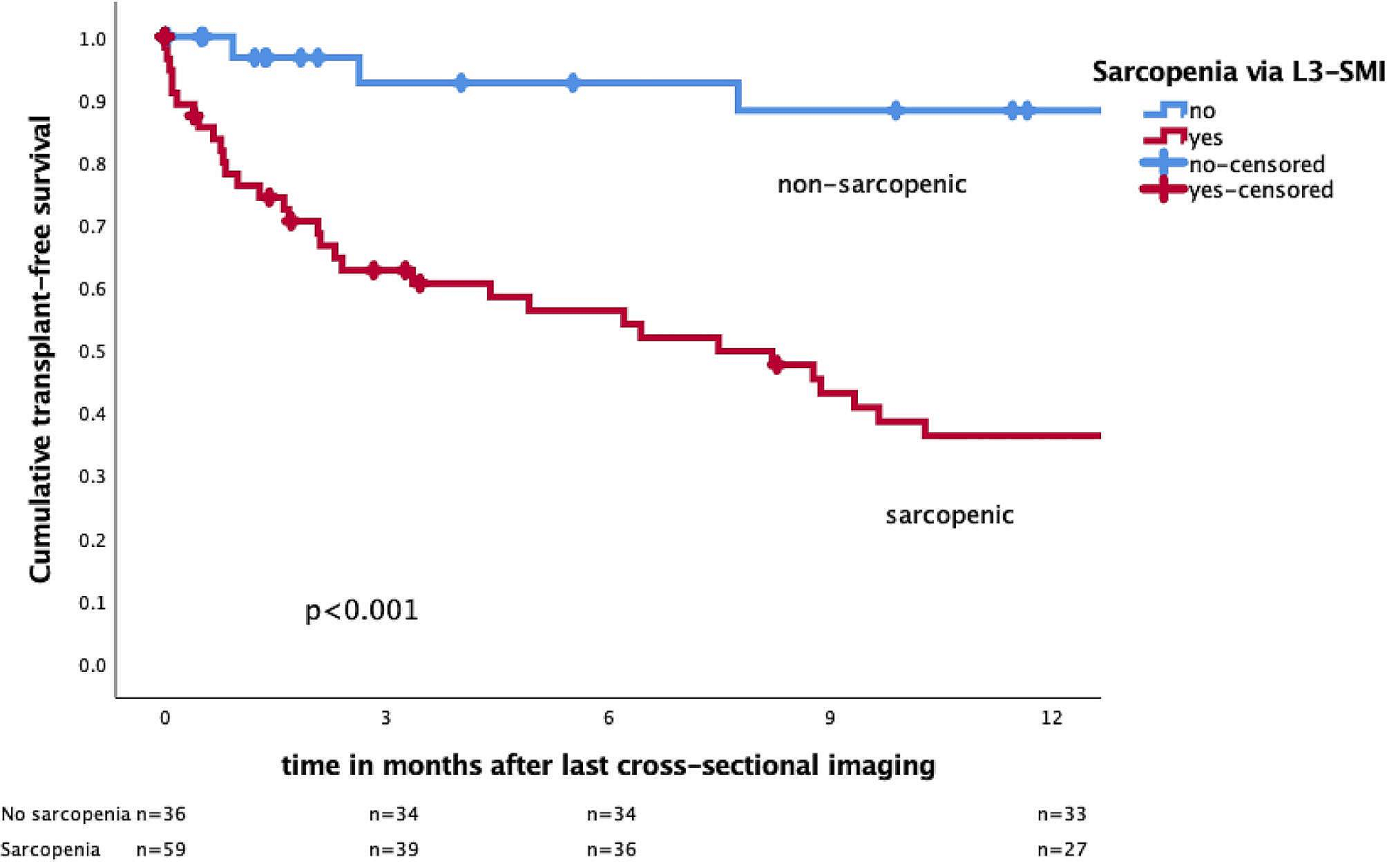
Survival at 12 months after imaging in patients with and without sarcopenia indicated by Kaplan-Meier curves Transplant-free survival of *n* = 95 patients with PSC at 12 months after last cross-sectional imaging relative to sarcopenia determined by skeletal muscle index at vertebra L3 (L3-SMI). Transplant-free survival 12 months after last imaging was at 91.6% for patients without sarcopenia compared to 45.8% of patients with detected sarcopenia (log-rank test *p* < 0.001). Censored patients shown as +

In patients with sarcopenia, only 45.8%, 39.0%, and 33.9% of patients reached TFS at 1, 2, and 5 years after the date of imaging, respectively, compared to 91.7%, 83.3%, and 83.3% in patients without sarcopenia (*p* < 0.001, log-rank test; Fig. 2-3) . Stratified by the presence of sarcopenia we observed that almost half of the sarcopenic patients, 47.5% (*n* = 28/59) received LTX, in comparison to only 16.7% in patients without sarcopenia (*n* = 6/36). All deceased patients were found to have had sarcopenia through imaging-based assessment of L3-SMI, representing a mortality rate of 25.4% in the sarcopenic group. No deaths were recorded in the non-sarcopenic patient group (*p* < 0.001). A significant difference in the estimated cumulative incidence between death (*p* = 0.011) and LTX (*p* = 0.008; competing risk) by the Fine-Gray test was observed at 5 years of imaging (Supplementary Fig. 2).

### Sarcopenia & sex-specific survival

We further investigated sex differences in the predictive value of sarcopenia. In the male patient group, the 5-year TFS was 28.6% (*n* = 12/42) for sarcopenic patients in comparison to 81.8% (*n* = 18/22) for patients without sarcopenia (log-rank test *p* < 0.001). In the female cohort, 47.1% (*n* = 8/17) of patients with sarcopenia reached TFS at 5 years, as opposed to 85.7% (*n* = 12/14) of the female patients without sarcopenia (log-rank test *p* = 0.043, Supplementary Figs. 3 and 4).

### Sarcopenia & BMI in SE criteria

In our study, SE status was assigned to 27.4% (*n* = 26) of patients according to German MELD SE criteria (2022) for PSC. Nearly half of the patients (46.2%; *n* = 12 of 26) received SE status because they met the criterion of BMI-reduction (Supplementary Table 4). Sarcopenia was identified in 84.6% (*n* = 22 of 26) of patients with SE status.

Based on the present results, we substituted BMI reduction (Table 2) with L3-SMI sarcopenia to create a modified set of SE criteria as a variable for statistical analysis. To facilitate comparison, the former German listing criteria will be referred to as SEG (**S**tandard **E**xception criteria **G**ermany), whereas the modified (M) version, including sarcopenia, will be referred to as M-SEG. The newly released German SE criteria for 2023 are referred to as the New SEG23. An extended comparison Table of the SE criteria is provided in the Supplementary Material (Supplementary Table 5). By applying the M-SEG criteria to the study cohort, 46.3% (*n* = 44) of patients would hypothetically be receiving M-SEG status.

SEG was compared to M-SEG to check for statistical differences in predicting TFS using log-rank tests and multivariable Cox regression models (additional analyses: Supplementary Tables 6–8 and supplementary Figs. 5–8).

**Fig. 4:**
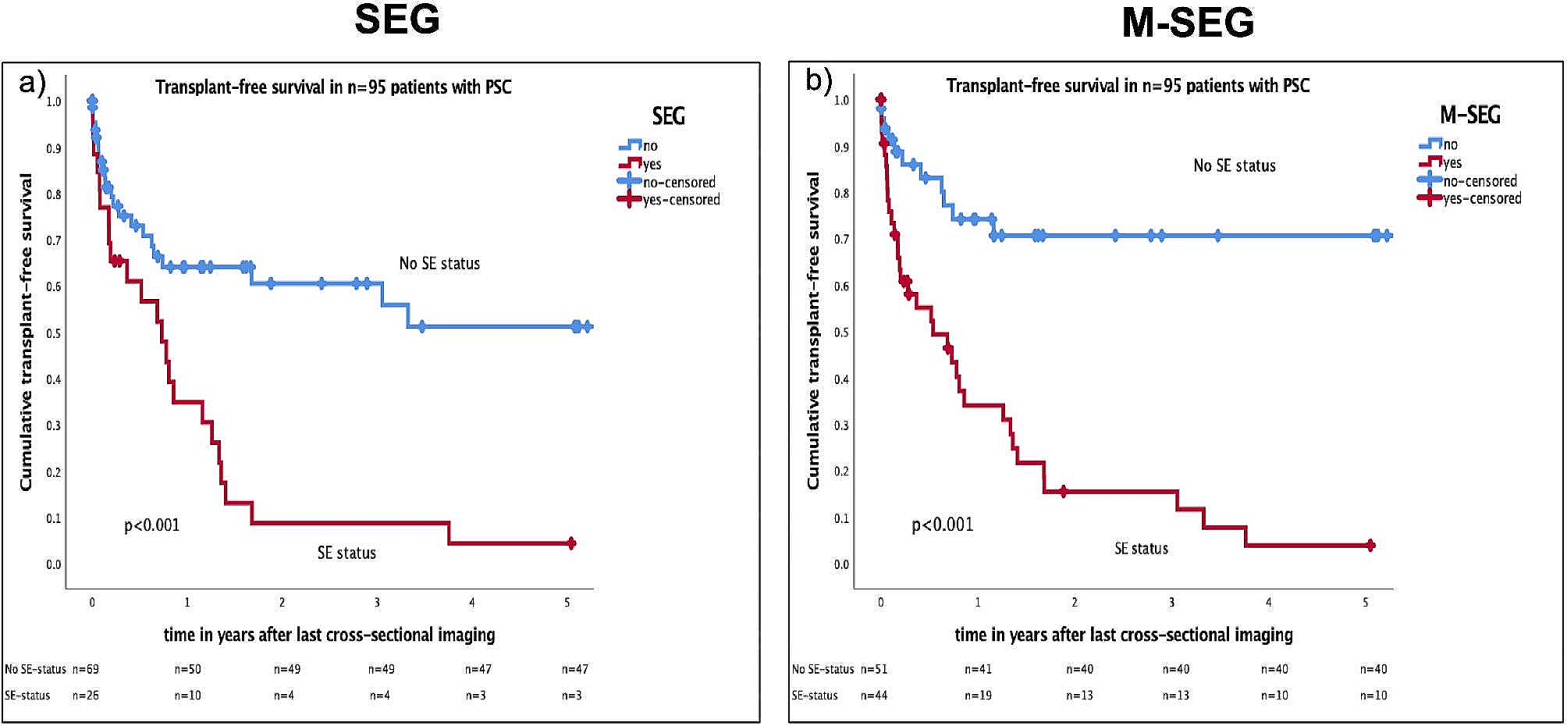
Comparison of five-year transplant-free survival after imaging in patients with PSC meeting German standard exception criteria (2022) versus modified criteria, indicated by Kaplan-Meier curves Juxtaposition of two Kaplan-Meier analyses depicting transplant-free survival (TFS) of *n* = 95 patients with PSC at 5 years after last cross-sectional imaging. Two different sets of MELD SE criteria for PSC were used to compare predictive accuracy regarding TFS: **a**) SEG (Standard Exception criteria Germany used until 12th September 2023) on the left side versus **b**) M-SEG (Modified-SEG) on the right side, which includes detection of sarcopenia via L3-SMI as third criterion instead of BMI reduction >10% in 12 months Log-rank test *p* < 0.001 for both analyses. Censored patients shown as + Results: **a**) TFS at 5 years of patients with SEG status was 11.5% versus 68.1% in patients without SE status **b**) TFS at 5 years of patients with M-SEG status was 22.7% versus 78.4% in patients without SE status

When comparing TFS in SEG and M-SEG by log-rank test, illustrated by the Kaplan-Meier curves in Fig. 4, a difference in survival rates was observed. In patients not meeting SE status, 68.1% (*n* = 47 of 69 patients) reached TFS at 5 years after cross-sectional imaging according to SEG, as opposed to 78.4% (40 of *n* = 51) when M-SEG criteria were applied.

Multivariable regression analysis revealed that M-SEG was significantly associated (*p* < 0.001) with 5-year TFS and was superior to SEG in predicting TFS (*p* = 0.141) (Table 3).

**Table 3:** Comparison between two sets of standard exception criteria predicting 5-year transplant-free survival by multivariable Cox regression analysis in patients with primary sclerosing cholangitis

Multivariate Cox regression model	B	SE	HR	95.0% CI for HR		*p*-value
Parameter				Lower	Upper	
SEG	0.525	0.357	1.691	0.841	3.402	0.141
M-SEG*	1.388	0.417	4.007	1.770	9.073	< 0.001

*M-SEG (Modified SE criteria Germany) includes dominant stenosis, recurring cholangitis ≥ 2 septic ep. in 6 months and sarcopenia via L3-SMI with cut-offs at < 50 cm2/m2 (for males) & <39 cm2/m2 (for females) which replaces BMI reduction of > 10% in 12 months

Abbreviations: M-SEG, Modified standard exception criteria Germany; SEG, standard exception criteria Germany for patients with PSC until 12th September 2023

## Discussion

Our study demonstrates that cross-sectional imaging-based L3-SMI sarcopenia represents an independent risk factor for transplant-free survival in patients with PSC. This finding supports the growing evidence that sarcopenia is an independent predictor of adverse outcomes in patients with other CLD [23].

The definition of sarcopenia has evolved rapidly throughout recent years. The new consensus definition considers sarcopenia to be a compound of loss of mass, quality, and strength of skeletal muscle tissue [7]. CT and MRI are the gold standards for quantitative muscle analysis in studies on cirrhosis [24, 25]. Clinical examination of extremity circumference, muscle strength measured by hand grip strength, or evaluation of muscle quality via ultrasound provide different approaches to assessment of sarcopenia but are time-consuming to perform [7, 26]. Conversely, diagnostic cross-sectional imaging is routinely performed during LTX assessments, providing opportunities for fast and reproducible measurements of body composition [11].

Muscle quantification methods specifically at the L3 vertebra level have shown a strong correlation with the whole-body muscle mass [27]. The skeletal muscle index (SMI) is widely used and well validated for prediction of mortality and other adverse outcomes in patients with CLD [20–22, 28]. Other commonly performed methods for muscle quantification at L3 include psoas muscle measurements such as transverse psoas muscle thickness (TPMT) or psoas muscle index (PMI) [9, 10, 29, 30]. However, the predictive value of PMI for outcomes of CLD remains controversial. Although some studies have observed a significant correlation between PMI and skeletal muscle mass measured by bioimpedance analysis, other studies have doubted the predictive value of PMI [31, 32]. Another study reported that PMI performed poorly in predicting mortality in a study of 396 patients with end-stage liver disease, whereas SMI was appropriately correlated with mortality [21]. Thus, L3-SMI is currently considered the more robust predictor of whole-body muscle mass [12, 21]. Therefore, we selected sarcopenia assessment via L3-SMI in this study, following current EASL guidelines.

To the best of our knowledge, this is the first study to examine transplant-free survival using imaging-based diagnosis of sarcopenia in patients with PSC. To date, little has been reported on the prognostic value of sarcopenia in PSC. In a retrospective study by Kikuchi et al., 22 patients with PSC were matched with a control group of 44 individuals free of hepatic disorders. This study examined the suitability of skeletal muscle mass as a prognostic marker [29]. By comparing, inter alia, SMI and PMI with clinical and laboratory data, they found that muscle mass was closely related to disease severity and prognosis of patients with PSC [29]. It should be emphasized that our study examined the effect of sarcopenia within a group of only patients with PSC, whereas Kikuchi et al. determined the effect in comparison to a healthy population [29].

By demonstrating that L3-SMI predicts adverse outcomes in an only PSC patient cohort, our findings support the idea that imaging-based detection of sarcopenia may be a useful tool for risk stratification that could potentially enhance the MELD standard exception criteria for PSC patients [28]. Therefore, we retrospectively assessed the impact of BMI on TFS in our cohort, as assessment of body composition via BMI reduction > 10% was part of the German SE criteria at the time and still is in other Eurotransplant member countries. In our multivariable Cox regression model, BMI reduction showed no significant correlation with TFS, in contrast to the L3-SMI sarcopenia.

Existing evidence on the impact of BMI reduction on the outcomes in patients with CLD is controversial [33, 34]. BMI is convenient and widely accepted, yet it is unable to comprehensively measure body composition as it poorly indicates the actual percentage or distribution of muscle and adipose tissue [35, 36]. Moreover, dynamic patient behavior before weight measurement, additional medical conditions, and fluid retention due to ascites may distort BMI measurements. Through the substitution of L3-SMI sarcopenia for BMI reduction, the M-SEG showed improved predictive accuracy in our multivariate Cox regression model. Our data highlight the importance of sarcopenia in patients with PSC. These results may be further relevant considering the similarity between the former German SE criteria and the current international Eurotransplant SE criteria. Further studies are necessary to assess the potential role of sarcopenia diagnosis in transplantation criteria. Recently, new German SE criteria have been implemented that neither include BMI, nor sarcopenia. Importantly, our study focused on the previously used German SE criteria since all patients were treated according to these criteria.

The Implementation of sarcopenia assessment in clinical scoring systems poses several challenges. The acquisition of special software to measure muscle mass and radiological experience comprise obstacles [18]. Furthermore, muscle quantification methods such as L3-SMI bear the risk of misinterpretation as muscle loss might be masked by changes in muscle architecture i.e. myosteatosis. Myosteatosis is defined as pathological infiltration of fat into muscle. This change in muscle composition is an indicator of poor muscle quality as the ratio of muscle mass to muscle strength worsens [37]. Myosteatosis may not necessarily occur simultaneously to the loss of muscle mass [38]. Thus far, it remains uncertain whether pathological fat accumulation in muscle is caused by loss of muscle mass or if is a distinct process that develops ahead of alterations in muscle mass [38]. Nonetheless there are various studies on the concurrent presence of these two muscle abnormalities. Prospective studies are required to investigate combined assessment of muscle quantity and quality to improve the diagnostic accuracy of sarcopenia.

Furthermore, imaging-based evaluation of sarcopenia for post-LTX management in patients with PSC represents another issue that requires further research.

Owing to its retrospective design, this study has several limitations. Selection bias in patients cannot be ruled out, as only patients with available cross-sectional images were included and only the latest imaging was used for measurements. The reliability of results on sex difference is impaired, as calculations are based on a total of 31 included female patients, owing to the specific sex ratio of PSC which is predominantly male. Due to the limited number of adequate imaging available, we were unable to obtain additional clinical data on muscle quality and strength relevant to further evaluate sarcopenia, such as the intramuscular fat fraction.

## Conclusions

In conclusion, cross-sectional imaging-based diagnosis of sarcopenia via L3-SMI was an independent predictor of transplant-free survival in this study of patients with PSC. The inclusion of L3-SMI sarcopenia into the modified SE criteria could potentially improve the prognostic accuracy of the SE criteria for patients with PSC. Future prospective, large-scale, and multicenter studies are required to corroborate our findings and further improve imaging-based diagnosis of sarcopenia in patients. Our study suggests that the imaging-based diagnosis of sarcopenia merits further scientific attention.

### Electronic supplementary material

Below is the link to the electronic supplementary material.

Supplementary Material 1

## Data Availability

The data underlying the findings of this study are available from the corresponding authors, upon reasonable request. Due to their containing information, data are not publicly available in order to protect the privacy of research participants.

## Declarations

### Presentation

Preliminary selected data of this study have previously been presented at the Viszeralmedizin 2022 Conference of the German Society for Digestive and Metabolic Diseases (DGVS) in Hamburg and the United European Gastroenterology (UEG) Week 2022 in Vienna.

## Abbreviations

BMI: body mass index
CCA: cholangiocarcinoma
CLD: chronic liver disease
CPG: clinical practice guidelines
CRC: colorectal carcinoma
EASL: the European Association for the Study of the Liver
ERCP: endoscopic retrograde cholangiopancreatography
ET: Eurotransplant
IBD: inflammatory bowel disease
INR: international normalized ratio
LTX: liver transplantation
L3-SMI: the third lumbar vertebra skeletal muscle index
MELD: Model of End-Stage Liver Disease
MRCP: magnetic resonance cholangiopancreatography
M-SEG: Modified standard exception criteria Germany
New SEG23: new German standard exception criteria since 12th September 2023
PMI: psoas muscle index
PSC: primary sclerosing cholangitis
SE: standard exception
SEG: standard exception criteria Germany until 12th September 2023
SMI: skeletal muscle index
TFS: transplant-free survival
TPMT: transverse psoas muscle thickness

## References

[1] Yu J, Refsum E, Helsingen LM (2022). Risk of hepato-pancreato-biliary cancer is increased by primary sclerosing cholangitis in patients with inflammatory bowel disease: a population-based cohort study. United Eur Gastroenterol J.

[2] Claessen MMH, Vleggaar FP, Tytgat KMAJ, Siersema PD, van Buuren HR (2009). High lifetime risk of cancer in primary sclerosing cholangitis. J Hepatol.

[3] Klose J, Klose MA, Metz C (2014). Outcome stagnation of liver transplantation for primary sclerosing cholangitis in the Model for End-Stage Liver Disease era. Langenbecks Arch Surg.

[4] Umgelter A, Hapfelmeier A, Kopp W, van Rosmalen M, Rogiers X, Guba M (2017). Disparities in Eurotransplant liver transplantation wait-list outcome between patients with and without model for end-stage liver disease exceptions. Liver Transpl.

[5] Wedd JP (2017). Model for end-stage liver disease exceptions: a common problem. Liver Transpl.

[6] Chapter. 5 ET Liver Allocation System (ELAS). Accessed December 25, 2022. https://www.eurotransplant.org/wp-content/uploads/2022/10/H5-ELAS-MELD-October-2022.pdf

[7] Cruz-Jentoft AJ, Bahat G, Bauer J (2019). Sarcopenia: revised European consensus on definition and diagnosis. Age Ageing.

[8] Tantai X, Liu Y, Yeo YH (2022). Effect of Sarcopenia on survival in patients with cirrhosis: a meta-analysis. J Hepatol.

[9] Praktiknjo M, Book M, Luetkens J, et al. Fat-Free muscle Mass in magnetic resonance imaging predicts Acute-on-chronic liver failure and survival in decompensated cirrhosis a he study of liver diseases t merican association for. Hepatology. 2018;67(3). 10.1002/hep.29602/suppinfo 10.1002/hep.29602 29059469

[10] Praktiknjo M, Clees C, Pigliacelli A, et al. Sarcopenia is associated with development of acute-on-chronic liver failure in decompensated liver cirrhosis receiving transjugular intrahepatic portosystemic shunt. Clin Transl Gastroenterol. 2019;10(4). 10.14309/ctg.0000000000000025 10.14309/ctg.0000000000000025 PMC6602782 30939488

[11] Ebadi M, Bhanji RA, Dunichand-Hoedl AR, Mazurak VC, Baracos VE, Montano-Loza AJ (2020). Sarcopenia severity based on computed tomography image analysis in patients with cirrhosis. Nutrients.

[12] Reichelt S, Pratschke J, Engelmann C, Neumann UP, Lurje G, Czigany Z. Body composition and the skeletal muscle compartment in liver transplantation: turning challenges into opportunities. American Journal of Transplantation. Published online. 2022. 10.1111/ajt.17089 10.1111/ajt.17089 35523584

[13] Merli M, Berzigotti A, Zelber-Sagi S (2019). EASL Clinical Practice guidelines on nutrition in chronic liver disease. J Hepatol.

[14] Son SW, Song DS, Chang UI, Yang JM. Definition of Sarcopenia in chronic liver disease. Life. 2021;11(4). 10.3390/life11040349 10.3390/life11040349 PMC8074027 33923561

[15] Neubekanntmachung der Richtlinie gem. § 16 Abs. 1 S. 1 Nrn. 2 und 5 TPG für die Wartelistenführung und Organvermittlung zur Lebertransplantation Allgemeiner Teil Besonderer Teil. Published online 2023. 10.3238/arztebl.2023.RiliOrgaWlOvLeberTx20230912

[16] Karlsen TH, Folseraas T, Thorburn D, Vesterhus M (2017). Primary sclerosing cholangitis– a comprehensive review. J Hepatol.

[17] Chazouilleres O, Beuers U, Bergquist A (2022). EASL Clinical Practice guidelines on sclerosing cholangitis. J Hepatol.

[18] Paternostro R, Lampichler K, Bardach C (2019). The value of different CT-based methods for diagnosing low muscle mass and predicting mortality in patients with cirrhosis. Liver Int.

[19] Lee CM, Kang BK, Kim M (2021). Radiologic definition of Sarcopenia in chronic liver disease. Life.

[20] Carey EJ, Lai JC, Wang CW (2017). A multicenter study to define Sarcopenia in patients with end-stage liver disease. Liver Transpl.

[21] Ebadi M, Wang CW, Lai JC (2018). Poor performance of psoas muscle index for identification of patients with higher waitlist mortality risk in cirrhosis. J Cachexia Sarcopenia Muscle.

[22] Topan MM, Sporea I, Dănilă M, et al. Impact of sarcopenia on survival and clinical outcomes in patients with liver cirrhosis. Front Nutr. 2021;8. 10.3389/fnut.2021.766451 10.3389/fnut.2021.766451 PMC8566695 34746216

[23] Tandon P, Montano-Loza AJ, Lai JC, Dasarathy S, Merli M (2021). Sarcopenia and frailty in decompensated cirrhosis. J Hepatol.

[24] Sinclair M, Gow PJ, Grossmann M, Angus PW (2016). Review article: Sarcopenia in cirrhosis - aetiology, implications and potential therapeutic interventions. Aliment Pharmacol Ther.

[25] Beaudart C, McCloskey E, Bruyère O (2016). Sarcopenia in daily practice: assessment and management. BMC Geriatr.

[26] Nijholt W, Scafoglieri A, Jager-Wittenaar H, Hobbelen JSM, van der Schans CP (2017). The reliability and validity of ultrasound to quantify muscles in older adults: a systematic review. J Cachexia Sarcopenia Muscle.

[27] Shen W, Punyanitya M, Wang ZM (2004). Total body skeletal muscle and adipose tissue volumes: estimation from a single abdominal cross-sectional image. J Appl Physiol.

[28] Montano-Loza AJ, Duarte-Rojo A, Meza-Junco J, et al. Inclusion of sarcopenia within MELD (MELD-Sarcopenia) and the prediction of mortality in patients with cirrhosis. Clin Transl Gastroenterol. 2015;6(7). 10.1038/ctg.2015.31 10.1038/ctg.2015.31 PMC4816259 26181291

[29] Kikuchi Y, Miyamori D, Kanno K, et al. Clinical utility of computed tomography-based evaluation of trunk muscles in primary sclerosing cholangitis. Jpn J Radiol Published Online May. 2022;7. 10.1007/s11604-022-01283-0 10.1007/s11604-022-01283-0 35523920

[30] Ohara M, Suda G, Kimura M (2020). Analysis of the optimal psoas muscle mass index cut-off values, as measured by computed tomography, for the diagnosis of loss of skeletal muscle mass in Japanese people. Hepatol Res.

[31] Rutten IJG, Ubachs J, Kruitwagen RFPM, Beets-Tan RGH, Olde Damink SWM, Van Gorp T (2017). Psoas muscle area is not representative of total skeletal muscle area in the assessment of Sarcopenia in ovarian cancer. J Cachexia Sarcopenia Muscle.

[32] Hamaguchi Y, Kaido T, Okumura S (2016). Proposal for new diagnostic criteria for low skeletal muscle mass based on computed tomography imaging in Asian adults. Nutrition.

[33] Yin Y, Li Y, Shao L, et al. Effect of body mass index on the prognosis of liver cirrhosis. Front Nutr. 2021;8. 10.3389/fnut.2021.700132 10.3389/fnut.2021.700132 PMC8417598 34490322

[34] Karagozian R, Bhardwaj G, Wakefield DB, Baffy G (2016). Obesity paradox in advanced liver disease: obesity is associated with lower mortality in hospitalized patients with cirrhosis. Liver Int.

[35] Nuttall FQ (2015). Body mass index: obesity, BMI, and health: a critical review. Nutr Today.

[36] Nishikawa H, Osaki Y. Liver cirrhosis: evaluation, nutritional status, and prognosis. Mediators Inflamm. 2015;2015. 10.1155/2015/872152 10.1155/2015/872152 PMC4606163 26494949

[37] Naimo MA, Varanoske AN, Hughes JM, Pasiakos SM. Skeletal muscle quality: a biomarker for assessing physical performance capabilities in young populations. Front Physiol. 2021;12. 10.3389/fphys.2021.706699 10.3389/fphys.2021.706699 PMC8376973 34421645

[38] Ebadi M, Tsien C, Bhanji RA, et al. Myosteatosis in cirrhosis: a review of diagnosis, pathophysiological mechanisms and potential interventions. Cells. 2022;11(7). 10.3390/cells11071216 10.3390/cells11071216 PMC8997850 35406780

