# Supplementary material for: Imaging-based diagnosis of sarcopenia for transplant-free survival in primary sclerosing cholangitis
Source: BMC Gastroenterol. 2024 Apr 25;24:145. doi: 10.1186/s12876-024-03232-9 (PMC11044284; doi:10.1186/s12876-024-03232-9)
Supplement: Supplementary file 1 — Supplementary Material 1 [file 12876_2024_3232_MOESM1_ESM.docx]

**Imaging based diagnosis of sarcopenia For transplant-free survival in primary sclerosing cholangitis – A cross-sectional study**

Pedram Keshoofi^1,*^, Philipp Schindler^2^, Florian Rennebaum^1^, Friederike Cordes^3^, Haluk Morgul^4^, Moritz Wildgruber^5^, Hauke S. Heinzow^6^, Andreas Pascher^4^, Hartmut H. Schmidt^7^, Anna Hüsing-Kabar^1^, Michael Praktiknjo^1^, Jonel Trebicka^1^, Leon L. Seifert^1,8,9,*^

1. Medical Clinic B, Department of Gastroenterology, Hepatology, Endocrinology, Infectiology, University Hospital Muenster, 48149 Muenster, Germany;
   [p_kesh02@uni-muenster.de](mailto:p_kesh02@uni-muenster.de) (P.K.); [florian.rennebaum@ukmuenster.de](mailto:florian.rennebaum@ukmuenster.de) (F.R.); [annehuesing@gmx.de](mailto:annehuesing@gmx.de) (A.H.-K.); [Michael.Praktiknjo@ukmuenster.de](mailto:Michael.Praktiknjo@ukmuenster.de) (M.P.); [Jonel.Trebicka@ukmuenster.de](mailto:Jonel.Trebicka@ukmuenster.de) (J.T.), [LeonLouis.Seifert@ukmuenster.de](mailto:LeonLouis.Seifert@ukmuenster.de) (L.L.S.)
2. Clinic for Radiology, University Hospital Muenster, 48149 Muenster, Germany;
   [Philipp.Schindler@ukmuenster.de](mailto:Philipp.Schindler@ukmuenster.de)
3. Medical Clinic II, Euregio Hospital Nordhorn, 48529 Nordhorn, Germany;
   [friederike.pott@gmail.com](mailto:friederike.pott@gmail.com)
4. Department for General, Visceral and Transplant Surgery, University Hospital Muenster, 48149 Muenster, Germany;
   [haluk.morguel@ukmuenster.de](mailto:haluk.morguel@ukmuenster.de) (H.M.); [andreas.pascher@ukmuenster.de](mailto:andreas.pascher@ukmuenster.de) (A.P.)
5. Department of Radiology, University Hospital LMU Munich, 81377 Munich, Germany;
   [Moritz.Wildgruber@med.uni-muenchen.de](mailto:Moritz.Wildgruber@med.uni-muenchen.de)
6. Department of Internal Medicine I, Krankenhaus der Barmherzigen Brüder, 54292 Trier, Germany;
   [h.heinzow@bbtgruppe.de](mailto:h.heinzow@bbtgruppe.de)
7. Department of Gastroenterology, Hepatology and Transplantation Medicine, University Hospital Essen, University of Duisburg-Essen, Hufelandstr. 55, 45147 Essen, Germany;
   [Hartmut.Schmidt@uk-essen.de](mailto:Hartmut.Schmidt@uk-essen.de)
8. The Rockefeller University Center for Clinical and Translational Science, 10065 New York, NY, United States of America
9. Laboratory of Virology and Infectious Disease, The Rockefeller University, 10065 New York, NY, United States of America
10. Correspondence: [p_kesh02@uni-muenster.de](mailto:p_kesh02@uni-muenster.de) (P.K.); [LeonLouis.Seifert@ukmuenster.de](mailto:LeonLouis.Seifert@ukmuenster.de) (L.L.S)
    Footnote: Leon Louis Seifert, MD, Medical Clinic B, Department of Gastroenterology, Hepatology, Endocrinology, Infectiology, University Hospital Muenster, Albert-Schweitzer-Campus 1, Bldg. A14, 48149 Muenster, Germany
    Current affiliation: The Rockefeller University Hospital, 1230 York Avenue, 10065 NY, USA.

**Supplemental Digital Content**

**FIGURE LEGENDS**

**Supplementary Figure 1 Two abdominal CT-scans at L3 of one patient during process of muscle area measurement for obtaining L3-SMI**

*L3-SMI corresponds to the total skeletal muscle area measured at the L3 cross-section, which is normalized by height (m) of the patient.*

*Description:*

- - 1. *left picture: contains traced area of left psoas muscle colored in blue.*
    2. *right picture: all skeletal muscles at L3 are traced. Blue= left psoas muscle; green= right psoas muscle; red= abdominal wall muscle and trunk muscle.*

**Supplementary Figure 2 Competing risk analysis with all-cause death and liver transplantation at 5 years after imaging in PSC patients with and without L3-SMI sarcopenia**

*Estimated cumulative incidence curves with all-cause mortality (1) and liver-transplantation (2) as competing events for a cohort of 95 patients with PSC, grouped by L3-SMI sarcopenia.
Gray’s Test for statistical difference of cumulative incidence curves:*

1. *p=0.011*
2. *p=0.008*

**Supplementary Figure 3 Kaplan-Meier curves demonstrating survival at 5 years after imaging in PSC patients with and without sarcopenia split by sex**

*Transplant-free survival (TFS) of n=95 patients with PSC at 5 years after last cross-sectional imaging relative to sarcopenia determined by skeletal muscle index at vertebra L3 (L3-SMI).
Patient group is split by sex for comparison with a) n=64 male patients and b) n=31 female patients.
Censored patients shown as +.*

*a) In male patient group, TFS 5 years after last imaging was 28.6% in patients with sarcopenia vs. 81.8% in patients without detected sarcopenia (log-rank test p<0.001).
b) In female patient group, TFS 5 years after last imaging was at 47.1% in patients with sarcopenia vs. 85.7% in patients without detected sarcopenia (log-rank test p=0.043).*

**Supplementary Figure 4 Kaplan-Meier curves demonstrating survival at 12 months after imaging in PSC patients with and without sarcopenia split by sex**

*Transplant-free survival (TFS) of n=95 patients with PSC at 12 months after last cross-sectional imaging relative to sarcopenia determined by skeletal muscle index at vertebra L3 (L3-SMI). Patient group is split by sex for comparison with a) n=64 male patients and b) n=31 female patients.*

*Censored patients shown as +.*

*a) In male patient group, TFS 12 months after last imaging was 42.9% in patients with sarcopenia vs. 95.5% in patients without detected sarcopenia (log-rank test p<0.001).
b) In female patient group, TFS 12 months after last imaging was at 52.9% in patients with sarcopenia vs. 85.7% in patients without detected sarcopenia (log-rank test p=0.108).*

**Supplementary Figure 5 Comparison of survival at 12 months after imaging in PSC patients meeting German SE criteria (2022) versus modified SE criteria, indicated by Kaplan-Meier curves**

*Juxtaposition of two Kaplan-Meier analyses depicting transplant-free survival (TFS) of n=95 patients with PSC at 12 months after last cross-sectional imaging. Two different sets of MELD SE criteria for PSC were used to compare predictive accuracy regarding TFS: a) SEG (Standard Exception criteria Germany) on the left side versus b) M-SEG (Modified-SEG) on the right side, which includes detection of sarcopenia via L3-SMI as third criterion instead of BMI reduction. Log-rank test p=0.019 for SEG and p=0.001 for M-SEG.*

*Censored patients shown as +.*

*Results:
a) TFS at 12 months of patients with SEG status was 38.5% versus 72.5% in patients without SE status.*

*b) TFS at 12 months of patients with M-SEG status was 43.2% versus 80.1% in patients without SE status.*

**Supplementary Figure 6 Comparison of survival at 5 years after imaging in PSC patients grouped by EASL CPG 2022 risk stratification approach versus modified SE criteria (M-SEG), indicated by Kaplan-Meier curves**

*Juxtaposition of two Kaplan-Meier analyses depicting transplant-free survival (TFS) of n=95 patients with PSC at 5 years after last cross-sectional imaging to compare predictive accuracy.*

*Left side a): Patient cohort grouped for comparison via criteria of EASL clinical practice guidelines 2022 approach to simple risk stratification.*

*TFS at 5 years of patients with “Low Risk” was 80.9% versus 44.6% in patients with “Significant Risk” (Log rank test p=0.005).*

*Right side b): Patient cohort grouped for comparison by M-SEG (Modified Standard Exception criteria Germany) which includes detection of sarcopenia via L3-SMI as third criterion instead of BMI reduction.*

*TFS at 5 years of patients with M-SEG status was 22.7% versus 78.4% in patients without SE status (Log rank test p<0.001).*

*Censored patients shown as +.*

*Results:
a) TFS at 5 years of patients with “Low Risk” was 80.9% versus 44.6% in patients with “Significant Risk” (Log rank test p=0.005).*

*b) TFS at 5 years of patients with M-SEG status was 22.7% versus 78.4% in patients without SE status (Log rank test p<0.001).*

**

**Supplementary Figure 7 Comparison of survival at 5 years after imaging in PSC patients grouped by modified SE criteria (M-SEG) versus New German Standard Exception criteria of 2023 (New SEG23) indicated by Kaplan-Meier curves**

*Juxtaposition of two Kaplan-Meier analyses depicting transplant-free survival (TFS) of n=95 patients with PSC at 5 years after last cross-sectional imaging. Two different sets of SE criteria for PSC were used to compare predictive accuracy regarding TFS:
a) Modified Standard Exception criteria Germany (M-SEG) including recurring cholangitis, dominant stenosis, and sarcopenia via L3-SMI.*

*b) New Standard Exception criteria Germany since January 2023 (New SEG23), including*

*significant bile duct stricture and elevated bilirubin >6 mg/dl during six months.
Log-rank test p<0.001 for both analyses. Censored patients shown as +.*

*Results:
a) TFS at 5 years of patients with M-SEG status was 22.7% versus 78.4% in patients without SE status.*

*b) TFS at 5 years of patients with new SEG23 status was 08.3% versus 67.6% in patients without SE status.*

**Supplementary Figure 8 Comparison of survival at 12 months after imaging in PSC patients grouped by modified SE criteria (M-SEG) versus New German Standard Exception criteria of 2023 (New SEG23) indicated by Kaplan-Meier curves.***Juxtaposition of two Kaplan-Meier analyses depicting transplant-free survival (TFS) of n=95 patients with PSC at 12 months after last cross-sectional imaging. Two different sets of SE criteria for PSC were used to compare predictive accuracy regarding TFS:
a) Modified Standard Exception criteria Germany (M-SEG) including recurring cholangitis, dominant stenosis, and sarcopenia via L3-SMI.
b) New Standard Exception criteria Germany since January 2023 (New SEG23), including significant bile duct stricture and elevated bilirubin >6 mg/dl during six months.
Log-rank test p<0.001 for both analyses. Censored patients shown as +.
Results:
a) TFS at 12 months of patients with M-SEG status was 43.2% versus 80.1% in patients without SE status.
b) TFS at 12 months of patients with M-SEG status was 16.6% versus 78.9% in patients without SE status.*

**Supplementary Figure 9** **Impact of sex as comparing factor on transplant-free survival over 5 years after imaging within sarcopenic patients.**

*Transplant-free survival of n=59 sarcopenic patients with PSC at 5 years after last cross-sectional imaging. Transplant-free survival 5 years after last imaging was at 28.6% for male patients compared to 47.1% for female patients (log-rank test p=0.276).
Censored patients shown as +.*

**
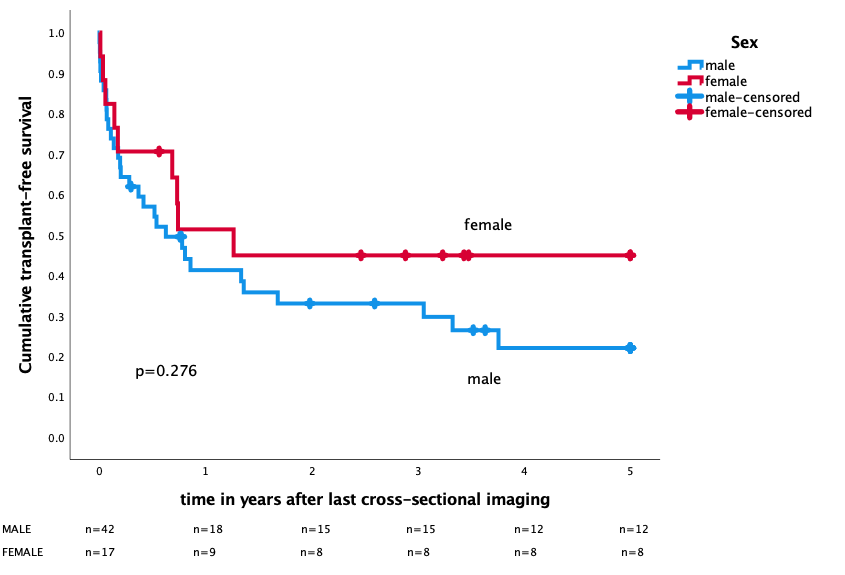
**

**Supplementary Figure 10** **Impact of liver cirrhosis as comparing factor on transplant-free survival over 5 years after imaging within sarcopenic patients.**

*Transplant-free survival of n=59 sarcopenic patients with PSC at 5 years after last cross-sectional imaging. Transplant-free survival 5 years after last imaging was at 11.1% for patients with liver cirrhosis compared to 69.6% for female patients (log-rank test p<0.001).
Censored patients shown as +.*

*
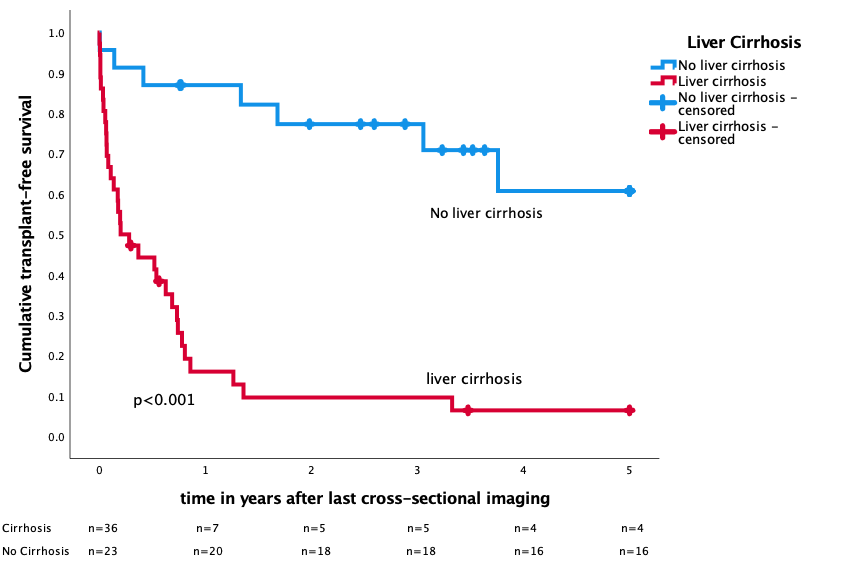
*

**Supplementary Figure 11** **Impact of liver decompensation as comparing factor on transplant-free survival over 5 years after imaging within sarcopenic patients.**

*Transplant-free survival of n=59 sarcopenic patients with PSC at 5 years after last cross-sectional imaging. Liver decompensation defined by presenting clinical features including ascites, hepatic encephalopathy or variceal hemorrhage. Transplant-free survival 5 years after last imaging was at 15.0% for patients with liver decompensation compared to 73.3% for patients without (log-rank test p<0.001). Censored patients shown as +.*

*
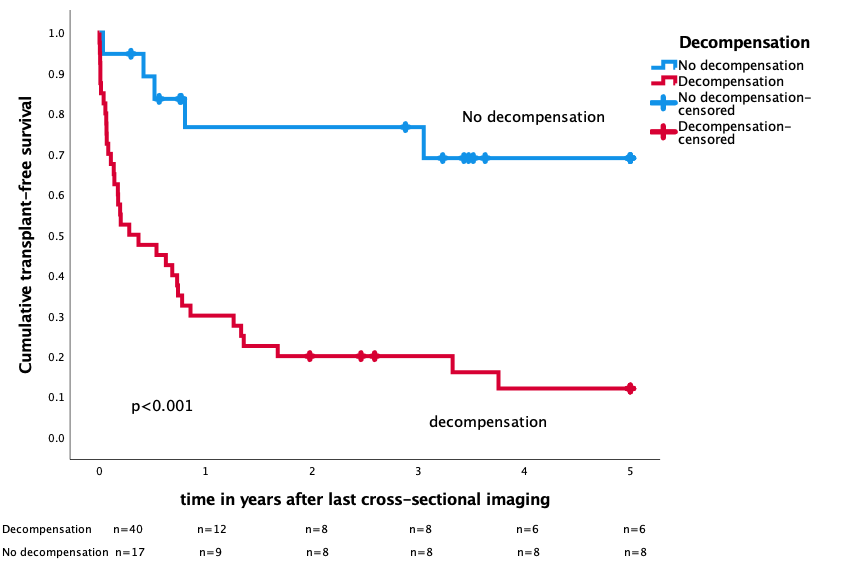
*

**Supplementary Figure 12** **Impact of sex as comparing factor on survival over 5 years after imaging within sarcopenic patients.**

*Survival of n=59 sarcopenic patients with PSC at 5 years after last cross-sectional imaging. Death is the single endpoint. Transplant-free survival 5 years after last imaging was at 88.1% for male patients compared to 64.7% for female patients (log-rank test p=0.103).
Censored patients shown as +.*

**
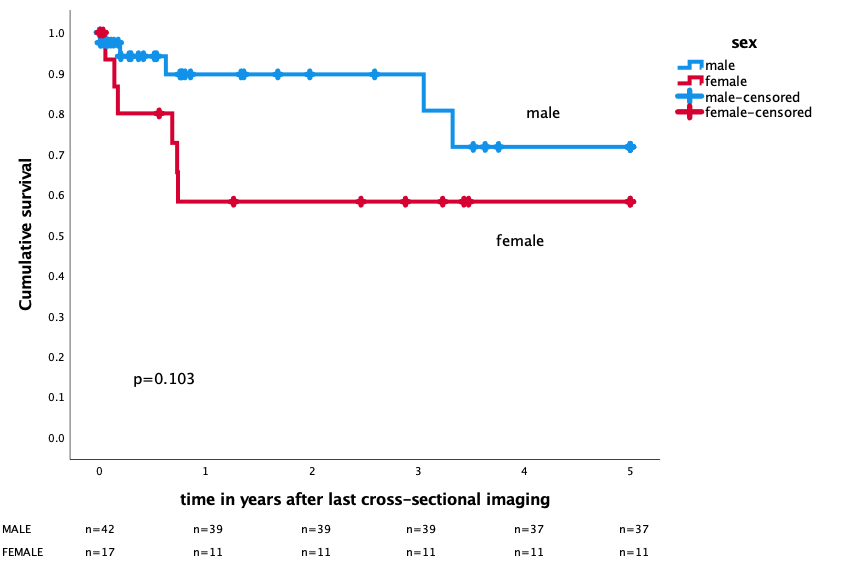
**

**Supplementary Figure 13** **Impact of liver cirrhosis as comparing factor on survival over 5 years after imaging within sarcopenic patients.**

*Survival of n=59 sarcopenic patients with PSC at 5 years after last cross-sectional imaging. Death is the single endpoint. Transplant-free survival 5 years after last imaging was at 75.0% for patients with cirrhosis compared to 91.3% for patients without cirrhosis (log-rank test p=0.004). Censored patients shown as +.*

*
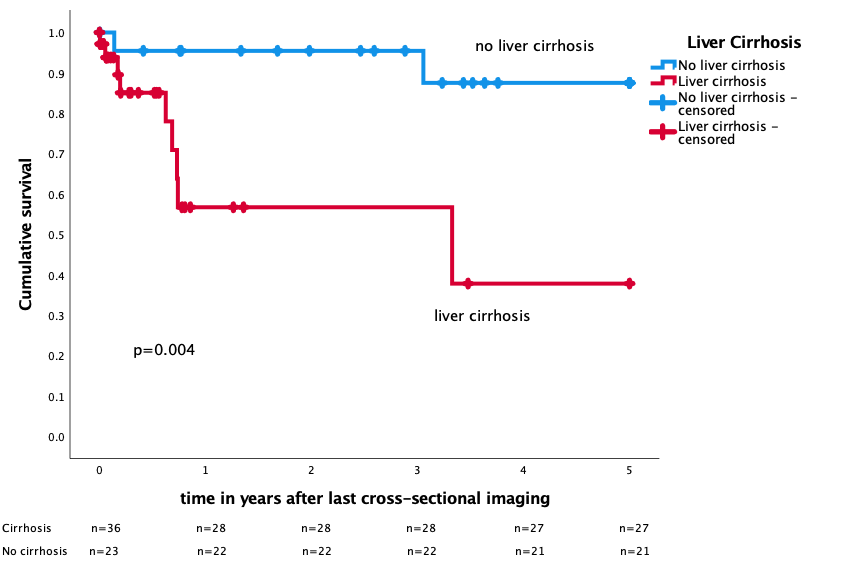
*

**Supplementary Figure 14** **Impact of liver decompensation as comparing factor on survival over 5 years after imaging within sarcopenic patients.**

*Transplant-free survival of n=59 sarcopenic patients with PSC at 5 years after last cross-sectional imaging. Liver decompensation defined by presenting clinical features including ascites, hepatic encephalopathy or variceal hemorrhage. Transplant-free survival 5 years after last imaging was at 75.0% for patients with liver decompensation compared to 94.7% for patients without (log-rank test p<0.001). Censored patients shown as +.*

**
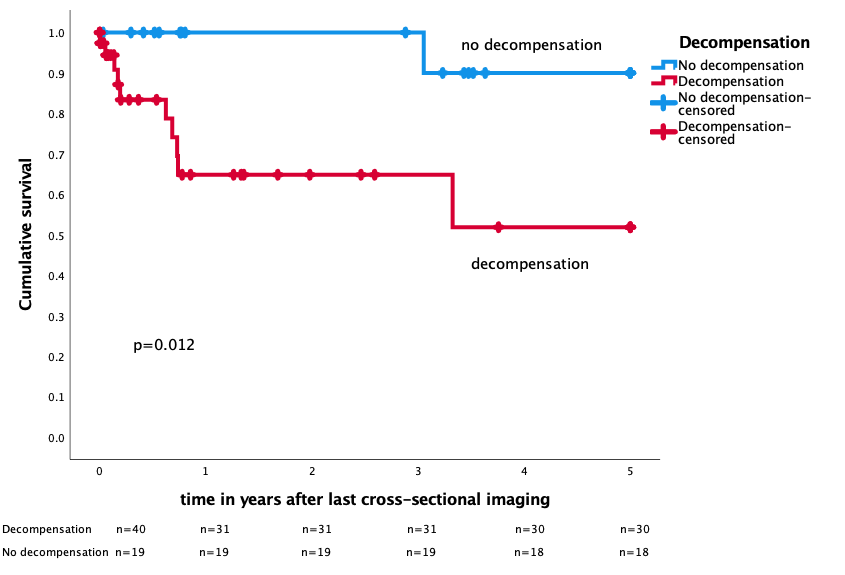
**

**TABLES**

**Supplementary Table 1** Baseline characteristics (EXTENDED VERSION)

| Parameters | All Patients | Sarcopenia | No-sarcopenia | *p*-value |
| --- | --- | --- | --- | --- |
| N= available cases in () if values are missing | % (total number) or median/mean (range/SD) | | |  |
| Number of patients | 95 | 62.1% (59) | 37.9% (36) | – |
| Sex |  |  |  | 0.310 |
| Male | 67.4% (64) | 71.2% (42) | 61.1% (22) |  |
| Female | 32.6% (31) | 28.8% (17) | 38.9% (14) |  |
| Age (median, range, in years) | 45 (18-76) | 46 (18-76) | 41 (23-64) | 0.369 |
| Height (m) | 1.79 (0.10) | 1.79 (0.11) | 1.78 (0.09) | 0.376 |
| Weight (kg) | 82.0 (24.6) | 80.1 (26.2) | 85.2 (21.7) | 0.336 |
| BMI | 25.7 (7.7) | 24.8 (8.1) | 27.1 (6.9) | 0.167 |
| PSC-IBD |  |  |  | 0.226 |
| Yes | 70.5% (67) | 66.1% (39) | 77.8% (28) |  |
| UC | 60.0% (57) | 55.9% (33) | 66.7% (24) |  |
| CD | 10.5% (10) | 10.2 % (6) | 11.1% (4) |  |
| No | 29.5% (28) | 33.4% (20) | 22.2% (8) |  |
| Liver cirrhosis |  |  |  | 0.017 |
| Yes | 51.6% (48) | 61.0% (36) | 35.3% (12) |  |
| No | 48.4% (45) | 39.0% (23) | 64.7% (22) |  |
| LTX |  |  |  | 0.002 |
| Yes | 35.8% (34) | 47.5% (28) | 16.7% (6) |  |
| No | 64.2% (61) | 52.5% (31) | 83.3% (30) |  |
| Death |  |  |  | 0.006 |
| Yes | 11.6% (11) | 18.6% (11) | 0% (0) |  |
| No | 84.2% (80) | 74.6% (44) | 100% (36) |  |
| LTX/death (primary endpoint) |  |  |  | <0.001 |
| Yes | 47.4% (45) | 66.1% (39) | 16.7% (6) |  |
| No | 52.6% (50) | 33.9% (20) | 83.3% (36) |  |
| Malignancy |  |  |  | 0.091 |
| Yes | 20.0% (19) | 25.4% (15) | 11.1% (4) |  |
| HCC | 1.1% (1) | 0% (0) | 2.8% (1) |  |
| CCC | 10.5% (10) | 15.3% (9) | 2.8%% (1) |  |
| CRC | 5.3% (5) | 6.8% (4) | 2.8% (1) |  |
| Other Carcinoma | 2.1% (2) | 1.7% (1) | 2.8% (1) |  |
| Combined | 1.1% (1) | 1.7% (1) | 0% (0) |  |
| No | 80.0% (76) | 74.6% (44) | 88.9% (32) |  |
| Smoking (n=83) |  |  |  | 0.850 |
| Yes | 15.7% (13) | 15.1% (8) | 16.7% (5) |  |
| No | 84.3% (70) | 84.9% (45) | 83.3% (25) |  |
| Dominant stenosis |  |  |  | 0.572 |
| Yes | 67.4% (64) | 69.5% (41) | 61.9% (23) |  |
| No | 32.6% (31) | 30.5% (18) | 36.1% (13) |  |
| Esophageal varices |  |  |  | 0.214 |
| Yes | 44.2% (42) | 49.2% (29) | 36.1% (13) |  |
| No | 55.8% (53) | 50.8% (30) | 63.9% (23) |  |
| Ascites |  |  |  | <0.001 |
| Yes | 40.0% (38) | 54.2% (32) | 16.7% (6) |  |
| No | 60.0% (57) | 45.8% (27) | 83.3% (30) |  |
| Hepatic Encephalopathy |  |  |  | 0.236 |
| Yes | 13.7% (13) | 16.9% (10) | 8.3% (3) |  |
| No | 86.3% (82) | 83.1% (49) | 91.7% (33) |  |
| Splenomegaly |  |  |  | 0.022 |
| Yes | 48.4% (46) | 57.6% (34) | 33.3% (12) |  |
| No | 51.6% (49) | 42.4% (25) | 66.7% (24) |  |
| Portal hypertensive gastropathy |  |  |  | 0.065 |
| Yes | 33.7% (32) | 40.7% (24) | 22.2% (8) |  |
| No | 66.3% (63) | 59.3% (35) | 77.8% (28) |  |
| BMI reduction >10% in 12 months |  |  |  | 0.083 |
| Yes | 16.8% (16) | 22.0% (13) | 8.3% (3) |  |
| No | 83.2% (79) | 78.0% (46) | 91.7% (33) |  |
| Recurring cholangitis (≥2x/6 months) |  |  |  | 0.083 |
| Yes | 16.8% (16) | 22.0% (13) | 8.3% (3) |  |
| No | 83.2% (79) | 78.0% (46) | 91.7% (33) |  |
| SEG (SE criteria of Germany) |  |  |  | 0.006 |
| Yes | 27.4% (26) | 37.3% (22) | 11.1% (4) |  |
| No | 72.6% (69) | 62.7% (37) | 88.9% (32) |  |
| MELD-Score (n=94) | 13.1 (8.2) | 14.9 (8.7) | 9.9 (6.2) | 0.002 |
| MELD-Sodium-score (n=93) | 14.2 (8.5) | 16.5 (9.0) | 10.3 (6.1) | <0.001 |
| Child-Pugh Grade (n=48) |  |  |  | 0.001 |
| A | 25.0% (12) | 13.9% (5) | 58.3% (7) |  |
| B | 50.0% (24) | 55.6% (20) | 33.3% (4) |  |
| C | 25.0% (12) | 30.6% (11) | 08.3% (1) |  |
| Mayo risk score (n=67) | 1.70 (1.87) | 2.27 (1.81) | 0.62 (1.52) | <0.001 |
| EASL 2022 CPG risk stratification |  |  |  | <0.001 |
| Low risk | 22.1% (21) | 15.3% (9) | 33.3% (12) |  |
| Significant risk | 77.9% (74) | 84.7% (50) | 66.7% (24) |  |
| HB (g/dl) | 12.2 (2.4) | 11.5 (2.5) | 13.4 (1.7) | <0.001 |
| Platelets (10^3^cells/μl) | 227 (149) | 231 (169) | 219 (110) | 0.708 |
| AP (units/l) (n=90) | 308 (178) | 327 (190) | 276 (155) | 0.188 |
| GGT (units/l) | 242 (385) | 259 (448) | 215 (255) | 0.592 |
| CRP (mg/dl) (n=83) | 3.0 (4.2) | 3.8 (4.5) | 1.4 (2.9) | 0.012 |
| GOT (median, range, in units/l) | 68 (20-765) | 84 (20-396) | 54 (20-765) | 0.528 |
| GPT (median, range, in units/l) | 63 (15-1190) | 61 (15-1190) | 76 (15-923) | 0.947 |
| Albumin (g/dl) (n=65) | 3.64 (0.86) | 3.36 (0.86) | 4.19 (0.57) | <0.001 |
| Bilirubin (mg/dl) | 5.0 (7.7) | 6.5 (8.8) | 2.7 (4.7) | 0.019 |
| INR (n=90) | 1.21 (0.41) | 1.29 (0.45) | 1.07 (0.32) | 0.012 |
| Creatinine (mg/dl) | 0.9 (0.5) | 0.9 (0.5) | 0.9 (0.6) | 0.855 |
| Sodium (mmol/l) (n=90) | 138 (4) | 137 (5) | 140 (2) | <0.001 |
| PTT (time in seconds) (n=90) | 38 (13) | 40 (12) | 35 (13) | 0.130 |
| Mode of cross-sectional imaging |  |  |  | 0.070 |
| CT | 58.9% (56) | 66.1% (39) | 47.2% (17) |  |
| MRI | 41.1% (39) | 33.9% (20) | 52.8% (19) |  |
| Time interval between diagnosis of PSC & date of last imaging (years) | 8.89 (7.52) | 8.95 (7.71) | 8.81 (7.32) | 0.929 |
| Loss to follow-up | 23.2% (22) | 15.3% (9) | 36.1% (13) | 0.019 |
| Duration of Loss to follow-up (years) | 1.29 | 1.04 (2.07) | 1.47 ( 1.91) | 0.620 |
| TSMA (cm^2^) | 135.66 (36.16) | 121.90 (30.94) | 157.33 (32.54) | <0.001 |
| SMI (cm²/m²) | 42.08 (9.35) | 37.55 (7.63) | 49.49 (6.31) | <0.001 |

Abbreviations: AP, alkaline phosphatase; BMI, body mass index; CCC, cholangiocarcinoma; CD, Crohn’s disease; CRC, colorectal cancer; CRP, C-reactive protein; CT, computed tomography; EASL 2022 CPG, the European Association for the Study of the Liver 2022 Clinical Practice Guidelines; GGT, Gamma-glutamyl transferase; GOT, glutamic oxaloacetic transaminase; GPT, glutamic-pyruvic transaminase; HB, hemoglobin; HCC, hepatocellular carcinoma; INR, international normalized ratio; LTX, liver transplantation; L3-SMI, the third lumbar vertebra skeletal muscle index; MELD score, Model of End-Stage Liver Disease score; MRI, magnetic resonance imaging; PSC-IBD, patient with primary sclerosing cholangitis and associated inflammatory bowel disease; PTT, partial thromboplastin time, SEG, standard exception criteria Germany until January 2023; SMI, skeletal muscle index; TSMA, total skeletal muscle area (at L3); UC, ulcerative colitis.

**Supplementary Table 2** Univariate Cox regression models of characteristics independently associated with predicting transplant-free survival at 5 years after last cross-sectional imaging (EXTENDED VERSION**)**

| Univariate Cox regression model |  |  |  | 95.0% CI HR | |  |
| --- | --- | --- | --- | --- | --- | --- |
| Parameter | B | SE | HR | Lower | Upper | *p*-value |
| Sarcopenia via L3-SMI | 1.716 | 0.440 | 5.561 | 2.346 | 13.182 | <0.001 |
| Sex | -0.499 | 0.347 | 0.607 | 0.307 | 1.198 | 0.150 |
| Age | 0.009 | 0.011 | 1.009 | 0.988 | 1.032 | 0.400 |
| BMI | 0.015 | 0.017 | 1.015 | 0.982 | 1.048 | 0.377 |
| PSC-IBD | -0.177 | 0.317 | 0.838 | 0.451 | 1.559 | 0.577 |
| Liver cirrhosis | 2.386 | 0.426 | 10.870 | 4.714 | 25.070 | <0.001 |
| Malignancy | 0.376 | 0.348 | 1.457 | 0.736 | 2.883 | 0.280 |
| Smoking | 0.371 | 0.418 | 1.449 | 0.639 | 3.287 | 0.374 |
| Dominant Stenosis | 1.075 | 0.392 | 2.930 | 1.359 | 6.317 | 0.006 |
| Esophageal Varices | 1.129 | 0.317 | 3.092 | 1.662 | 5.754 | <0.001 |
| Ascites | 1.876 | 0.329 | 6.528 | 3.426 | 12.442 | <0.001 |
| Hepatic Encephalopathy | 0.777 | 0.360 | 2.175 | 1.074 | 4.405 | 0.031 |
| Splenomegaly | 1.081 | 0.318 | 2.948 | 1.581 | 5.498 | <0.001 |
| Portal hypertensive gastropathy | 1.007 | 0.299 | 2.738 | 1.523 | 4.925 | <0.001 |
| BMI reduction >10% in 12 months | 1.133 | 0.327 | 3.106 | 1.636 | 5.894 | <0.001 |
| Recurring cholangitis (≥2x/6 months) | 0.997 | 0.329 | 2.711 | 1.424 | 5.161 | 0.002 |
| SEG (SE criteria of Germany) | 1.351 | 0.305 | 3.860 | 2.122 | 7.021 | <0.001 |
| MELD-score | 0.115 | 0.015 | 1.121 | 1.088 | 1.156 | <0.001 |
| MELD-sodium-score | 0.119 | 0.016 | 1.127 | 1.093 | 1.162 | <0.001 |
| Child-Pugh Grade | 1.316 | 0.246 | 3.727 | 2.302 | 6.036 | <0.001 |
| Mayo risk score | 0.463 | 0.093 | 1.589 | 1.325 | 1.905 | <0.001 |
| EASL 2022 CPG risk stratification | 1.362 | 0.525 | 3.904 | 1.396 | 10.919 | 0.009 |
| Hemoglobin | -0.392 | 0.063 | 0.676 | 0.597 | 0.765 | <0.001 |
| Platelets | -0.003 | 0.001 | 0.997 | 0.994 | 0.999 | 0.015 |
| AP | 0.000 | 0.001 | 1.000 | 0.999 | 1.002 | 0.526 |
| GGT | -0.001 | 0.001 | 0.999 | 0.998 | 1.000 | 0.157 |
| CRP | 0.101 | 0.023 | 1.106 | 1.057 | 1.158 | <0.001 |
| GOT | 0.001 | 0.001 | 1.001 | 0.999 | 1.003 | 0.444 |
| GPT | 0.000 | 0.001 | 1.000 | 0.998 | 1.002 | 0.977 |
| Albumin | -0.870 | 0.158 | 0.419 | 0.307 | 0.571 | <0.001 |
| Bilirubin | 0.108 | 0.017 | 1.114 | 1.078 | 1.151 | <0.001 |
| INR | 1.503 | 0.257 | 4.497 | 2.720 | 7.435 | <0.001 |
| Creatinine | 0.449 | 0.220 | 1.567 | 1.018 | 2.411 | 0.041 |
| Sodium | -0.112 | 0.024 | 0.894 | 0.852 | 0.938 | <0.001 |
| PTT | 0.038 | 0.007 | 1.039 | 1.025 | 1.054 | <0.001 |
| Mode of cross-sectional imaging | -1.074 | 0.348 | 0.342 | 0.173 | 0.675 | 0.002 |
| TSMA | -0.004 | 0.004 | 0.996 | 0.988 | 1.004 | 0.285 |
| SMI | -0.028 | 0.016 | 0.973 | 0.943 | 1.004 | 0.083 |

Abbreviations: AP, alkaline phosphatase; BMI, body mass index; CRP, C-reactive protein; EASL 2022 CPG, the European Association for the Study of the Liver 2022 Clinical Practice Guidelines; GGT, Gamma-glutamyl transferase; GOT, glutamic oxaloacetic transaminase; GPT, glutamic-pyruvic transaminase; INR, international normalized ratio; L3-SMI, the third lumbar vertebra skeletal muscle index; MELD score, Model of End-Stage Liver Disease score; PSC-IBD, patient with primary sclerosing cholangitis and associated inflammatory bowel disease; PTT, partial thromboplastin time, SEG, standard exception criteria Germany until January 2023; SMI, skeletal muscle index; TSMA, total skeletal muscle area (at L3)

**Supplementary Table 3** Characteristics associated with 5-year transplant-free survival by multivariable Cox regression analysis in patients with primary sclerosing cholangitis

| Multivariate Cox regression model |  |  |  | 95.0% CI HR | |  |
| --- | --- | --- | --- | --- | --- | --- |
| Parameter | B | SE | HR | Lower | Upper | *p*-value |
| Sarcopenia via L3-SMI | 1.011 | 0.459 | 2.749 | 1.118 | 6.758 | 0.028 |
| Dominant Stenosis | 0.212 | 0.446 | 1.237 | 0.516 | 2.961 | 0.634 |
| Splenomegaly | 0.906 | 0.368 | 2.473 | 1.203 | 5.087 | 0.014 |
| BMI reduction >10% in 12 months | 0.348 | 0.358 | 1.417 | 0.703 | 2.856 | 0.330 |
| Recurring cholangitis (≥2x/6months) | 0.928 | 0.385 | 2.529 | 1.190 | 5.376 | 0.016 |
| Bilirubin | 0.076 | 0.027 | 1.079 | 1.023 | 1.137 | 0.005 |
| INR | 0.445 | 0.429 | 1.560 | 0.673 | 3.617 | 0.300 |

Abbreviations: BMI, body mass index; INR, international normalized ratio; L3-SMI, the third lumbar vertebra skeletal muscle index.

**Supplementary Table 4** Characteristics of patients with regards to waiting list and liver transplantation

| Parameters | All Patients | Sarcopenia | No-sarcopenia | *p*-value |
| --- | --- | --- | --- | --- |
| N= available cases in () if values are missing | % (total number) or median/mean (range/SD) | | |  |
| Number of patients in total study cohort | 95 | 62.1% (59) | 37.9% (36) | – |
| Number of patients listed or with LTX | 54.7% (52) | 62.7% (37) | 41.7% (15) | 0.010 |
| 1) on waiting list | 18.9% (18) | 15.3% (9) | 25.0% (9) |  |
| Waiting list mortality | 27.8% (5) | 55.6% (5) | – |  |
| 2) received LTX | 35.8% (34) | 47.5% (28) | 16.7% (6) |  |
| Post-LTX mortality | 11.8% (4) | 14.2% (4) | – |  |
| Mean time (months) on waiting list  until LTX/ end of follow up | 28.9 | 29.1 | 28.4 | 0.928 |
| 1) on waiting list | 34.3 | 34.9 | 33.8 |  |
| 2) received LTX | 26.1 | 27.3 | 20.4 |  |
| SEG | 27.4% (26) | 37.3% (22) | 11.1% (4) | 0.006 |
| SE-status due to BMI-reduction   >10% in 1 year | 46.2% (12) | 45.5% (10) | 50.0% (2) |  |
| SE-status due to remaining two SE   criteria | 53.8% (14) | 54.5% (12) | 50.0% (2) |  |
| M-SEG | 46.3% (44) | – | – | – |
| SE-status due to L3-SMI sarcopenia | 68.2% (30) | – | – |  |

Abbreviations: BMI, body mass index; LTX, liver transplantation; L3-SMI, the third lumbar vertebra skeletal muscle index; SE, standard exception; M-SEG, Modified standard exception criteria Germany; SEG, standard exception criteria Germany for patients with PSC until January 2023.

**Supplementary Table 5** Comparison table of standard exception criteria for PSC including a modified version used in this study (M-SEG)

| **German SE criteria  for PSC 2022*** **- *SEG* -** | **Modified SE criteria  for PSC  *-* *M-SEG* -** | **New German SE criteria  for PSC since 2023 *-* New *SEG23* -** |
| --- | --- | --- |
| - ≥2 spontaneously occurring septic episodes with cholangitis within 6 months (not due to interventions, not treatable by interventions) - Documented development of dominant bile duct stenosis | | - Diagnosis of PSC by MRCP or ERCP with exclusion n of causes of secondary sclerosing cholangitis (SSC)  **AND** - Stricture of the common bile duct (DHC) and/or the lobular bile ducts (DHD,DHS)  **with** symptoms and signs of obstructive cholestasis with total bilirubin ≥ 6 mg/dl over 6 months, proven by MRCP or ERC and not amenable to interventional treatment. |
| - Body Mass Index reduction >10% within 12 months | - Imaging-based diagnosis of sarcopenia via skeletal muscle index at vertebra L3 with cut-off at:   - <50 cm^2^/m^2^ for males   - <39 cm^2^/m^2^ for females |  |
| **To receive SE points, PSC must be diagnosed according to standard radiology criteria and at least two country-dependent listing criteria must be met.*  Abbreviations: ERCP, endoscopic retrograde cholangiopancreatography; MRCP, magnetic resonance cholangiopancreatography; M-SEG, Modified standard exception criteria Germany; New SEG23, new German standard exception criteria since January 2023; SEG, standard exception criteria Germany for patients with PSC until January 2023. | | |

**Supplementary Table 6** Comparison between M-SEG and EASL CPG 2022 risk stratification approach predicting 5-year transplant-free survival by multivariable Cox regression analysis in patients with PSC

| Multivariate Cox regression model |  |  |  | 95.0% CI HR | |  |
| --- | --- | --- | --- | --- | --- | --- |
| Parameter | B | SE | HR | Lower | Upper | *p*-value |
| M-SEG* | 1.576 | 0.356 | 4.838 | 2.405 | 9.729 | <0.001 |
| EASL 2022 CPG risk stratification | 1.095 | 0.529 | 2.989 | 1.061 | 8.422 | 0.038 |

**M-SEG (Modified SE criteria Germany) includes dominant stenosis, recurring cholangitis ≥2 septic ep. in 6 months and sarcopenia via L3-SMI with cut-offs at <50 cm2/m2 (for males) & <39 cm2/m2 (for females) which replaces BMI reduction of >10% in 12 months.*

Abbreviations: M-SEG, Modified standard exception criteria Germany; the European Association for the Study of the Liver 2022 Clinical Practice Guidelines

**Supplementary Table 7** Comparison between M-SEG and the new German PSC SE criteria 2023 (New SEG23) predicting 5-year transplant-free survival by multivariable Cox regression analysis in patients with PSC

| Multivariate Cox regression model |  |  |  | 95.0% CI HR | |  |
| --- | --- | --- | --- | --- | --- | --- |
| Parameter | B | SE | HR | Lower | Upper | *p*-value |
| M-SEG* | 1.281 | 0.381 | 3.599 | 1.705 | 7.595 | <0.001 |
| New SEG23** | 1.512 | 0.338 | 4.536 | 2.338 | 8.801 | <0.001 |

**M-SEG (Modified SE criteria Germany) includes dominant stenosis, recurring cholangitis ≥2 septic ep. in 6 months and sarcopenia via L3-SMI with cut-offs at <50 cm2/m2 (for males) & <39 cm2/m2 (for females) which replaces BMI reduction of >10% in 12 months.
** New Standard Exception criteria Germany since January 2023 (New SEG23), including relevant bile duct stricture and elevated bilirubin >6 mg/dl during six months.*

Abbreviations: M-SEG, Modified standard exception criteria Germany; New SEG23, new German standard exception criteria since January 2023.

**Supplementary Table 8** between SEG, M-SEG and the new German PSC SE criteria 2023 (New SEG23) predicting 5-year transplant-free survival by multivariable Cox regression analysis in patients with PSC

| Multivariate Cox regression model |  |  |  | 95.0% CI HR | |  |
| --- | --- | --- | --- | --- | --- | --- |
| Parameter | B | SE | HR | Lower | Upper | *p*-value |
| SEG | 0.402 | 0.364 | 1.494 | 0.733 | 3.048 | 0.269 |
| M-SEG* | 1.060 | 0.439 | 2.885 | 1.221 | 6.819 | 0.016 |
| New SEG23** | 1.483 | 0.341 | 4.406 | 2.258 | 8.597 | <0.001 |

**M-SEG (Modified SE criteria Germany) includes dominant stenosis, recurring cholangitis ≥2 septic ep. in 6 months and sarcopenia via L3-SMI with cut-offs at <50 cm2/m2 (for males) & <39 cm2/m2 (for females) which replaces BMI reduction of >10% in 12 months.
** New Standard Exception criteria Germany since January 2023 (New SEG23), including relevant bile duct stricture and elevated bilirubin >6 mg/dl during six months.*

Abbreviations: M-SEG, Modified standard exception criteria Germany; New SEG23, new German standard exception criteria since January 2023; SEG, standard exception criteria Germany for patients with PSC until January 2023.

**Supplementary Table 9** Characteristics associated with 5-year transplant-free survival by multivariable Cox regression analysis in patients with primary sclerosing cholangitis

| Multivariate Cox regression model |  |  |  | 95.0% CI HR | |  |
| --- | --- | --- | --- | --- | --- | --- |
| Parameter | B | SE | HR | Lower | Upper | *p*-value |
| Sarcopenia via L3-SMI | 1.496 | 0.476 | 4.462 | 1.755 | 11.348 | 0.002 |
| MELD | 0.096 | 0.020 | 1.101 | 1.101 | 1.144 | <0.001 |
| Cirrhosis | 1.721 | 0.469 | 5.590 | 2.230 | 14.010 | <0.001 |
| Ascites | 0.569 | 0.369 | 1.767 | 0.858 | 3.641 | 0.123 |
| Sodium | 0.024 | 0.034 | 1.025 | 0.958 | 1.096 | 0.479 |

Abbreviations: L3-SMI, the third lumbar vertebra skeletal muscle index; MELD, Model of End-Stage Liver Disease

**Supplementary Table 10** Comparison between sarcopenia determined via skeletal muscle index at L3 and the MELD predicting 5-year transplant-free survival by multivariable Cox regression analysis in patients with PSC

| Multivariate Cox regression model |  |  |  | 95.0% CI HR | |  |
| --- | --- | --- | --- | --- | --- | --- |
| Parameter | B | SE | HR | Lower | Upper | *p*-value |
| Sarcopenia via L3-SMI | 1.435 | 0.443 | 4.200 | 1.763 | 10.003 | 0.001 |
| MELD | 0.112 | 0.018 | 1.119 | 1.081 | 1.158 | <0.001 |

Abbreviations: L3-SMI, the third lumbar vertebra skeletal muscle index; MELD, Model of End-Stage Liver Disease
